# Research on the characteristics of EV interior sound quality and its dynamic active control system design and development un-der accelerated driving conditions

**DOI:** 10.1371/journal.pone.0290150

**Published:** 2024-04-01

**Authors:** Shuai Zhang, Yipeng Li, Hang Jiang, Feifei He, Liyou XU, Feng Xiong, Yuntao Cao

**Affiliations:** 1 College of Vehicle and Traffic Engineering, Henan University of Science and Technology, Luoyang, China; 2 College of Vehicle Engineering, Chongqing University of Technology, Chongqing, China; 3 General R&D Institute of China FAW Group Co., Ltd., Changchun, China; 4 State Key Laboratory of Comprehensive Technology on Automobile Vibration and Noise & Safety Control, Changchun, China; Ankara University: Ankara Universitesi, TURKEY

## Abstract

In order to improve the interior sound quality of Electric Vehicles (EV), solve the problem of low sense of power and comfort of the interior sound as well as the large electromagnetic excitation order noise of motor and the sharp interior sound, this article designs a dynamic active sound control system for EV under accelerated driving conditions. Firstly, by comparing and analyzing the sound spectrum characteristics of fuel vehicle (FV) and EV during acceleration, a short-time Fourier transform (STFT) is adopted to extract and synthesize the engine sound. Secondly, the influence of the engine order composition and the energy distribution in the frequency domain on the sound quality of the vehicle is analyzed, and an active control system for sound quality is proposed. And the software and hardware development of the active control sound system is completed. Finally, through real-vehicle testing and verification, the sense of comfort and power of the EV interior sound has been greatly improved during acceleration, and the total value of interior sound can meet the requirement. The sound pressure level and loudness of interior sound have been increased, and the sharpness of the sound inside the vehicle has been improved, with a maximum reduction of 1.0acum.

## 1. Introduction

Vehicles has a lot of performance, lightweight and NVH performance is very important vehicle quality [1, 2]. With the development of vehicle technology, its NVH (noise, vibration, hardness) performance is one of the attributes most easily perceived by customers. In terms of vehicle driving sound design, some automobile brands have formed a distinct brand sound image after decades of accumulation [3]. For fuel vehicle (FV), NVH engineers can optimize the structure of inlet and exhaust or do research on engine sound to create a driving experience full of power and comfort from the perspective of sound [4, 5].

Vehicle lightweighting increases fuel economy on the basis of vehicle weight reduction [6, 7]. Due to the increasingly strict legal requirements on fuel economy and emission standards, more car companies focus on the research and development of new energy vehicles. For pure electric vehicles, due to the difference in structure between them and FV, there is no engine sound in driving, and motor high-frequency noise, road noise and wind noise are the main sound in dynamic driving. The monotonous electromagnetic excitation order sound of the motor causes problems such as loud noise when driving, sharp sound inside the vehicle, and serious homogenization of internal sound of different brands of electric vehicles when driving.

Active Noise Cancellation (ANC) technology is widely used to improve the internal sound quality of EVs. Son G H et al. [8] optimized the shape design of the gearbox housing to reduce the radiated noise of the gearbox of agricultural electric vehicles. Huang H B et al. [9] proposed a new noise source identification method based on interval analysis to identify and reduce the noise generated by the suspension structure of electric vehicles. Furqani J et al. [10] analyzed and tested a certain type of switched reluctance motor and verified that adopting new current waveform could reduce noise. Literature [11–13] has modeled and suppressed motor noise, providing theoretical basis and analytical method for noise prediction, optimization and evaluation of motors. Literature [14, 15] have studied and controlled the noise of hub-type motor. Kim B et al. [16] determined the noise source of the in-wheel motor system and proposed the low-noise design criteria through structural modification.

In addition, Heo H [17] used acoustic metal layers with high reflective properties and relatively light materials to effectively suppress the noise of automobile tires when driving. Cao Y et al. [18] studied the generation mechanism and frequency characteristics of intake noise of battery cooling system of electric vehicle, reduced intake noise by placing sound-absorbing materials in ventilation pipes, and verified its good noise reduction effect through subjective evaluation. Lee J W et al. [19] compared the sound absorption and sound insulation properties of the fiber cross-section shape of non-woven felt, indicating that the sound insulation properties mainly depended on the weight and stiffness of the specimen. Kim M W et al. [20] improved the sound absorption coefficient of the vehicle and reduced the radiated noise of the engine through material selection and layer optimization. Polyurethane foam has been widely used as automobile sound absorbing material due to its high sound absorbing efficiency. In [21, 22], this kind of sound absorbing material has been studied and analyzed.

However, customer expectations for NVH optimization for pure electric vehicles remain the same as for conventional vehicles. The most effective and widely used solution in vehicle is active sound generation technology, which uses additional sound systems to produce artificial engine sounds to make the vehicle more sense of sporty and comfortable. [5] Many scholars have proposed different methods for engine sound extraction and fitting. Baldan S et al. [23] proposed an engine sound fitting method based on four-stroke operation mechanism with simplified operation, established a mathematical model of engine sound based on this method, and verified the fitting accuracy of the model through spectral comparison with the actual tested engine sound. Patrick B et al. [24, 25] described in detail the implementation process of engine sound fitting based on digital audio control algorithm, and conducted sound fitting based on real-time vehicle operation parameter information. Min D K [26] proposed a method of engine sound fitting based on sine wave synthesis algorithm to represent the sound characteristics of the engine in a real and natural way. Park H W et al. [27] from Soongsil University in South Korea put forward an active sound generation method for engine sound, and the active sound generation system (ASGS) built based on this method can truly restore engine sound characteristics under the condition of occupying less hardware resources.

For ASGS, Lee S et al. [28–30] proposed an artificial neural network-based calculation method for engine rumble sensation index and low down sensation index. Meanwhile, they also proposed an active noise reduction control method based on AOF_LMS algorithm to control the amplitude of engine order sound in local speed range which realize the active design of engine order sound [31, 32]. Ahrens D et al. [33] proposed the concept of active sound management based on sound system, which can actively reduce the noise of the prominent part of the order component of the engine and actively enhance the sunken part of the order component. Park D et al. [34] developed a personalized engine sound active enhancement system, which can customize the development of optimal engine sound. Ryu S et al. [35] proposed a new algorithm for using virtual error microphone (VEM) to actively control the sound quality of automobile cockpit, which can reduce the degradation effect caused by the distance between the driver’s ear and the error microphone, so that the active voice system can provide more accurate sound. Utyuzhnikov S V et al. [36] proposed an active voice system based on real-time control algorithm, which makes the sound inside the car more comfortable by setting a secondary sound source on the boundary. Maunder M et al. [37, 38] analyzed the relationship between sound pressure level (SPL) and vehicle speed and pedal displacement when driving, providing a basis for the sound gain of active vocal system. Lee D Y et al. [39] studied the sound design method for acceleration in electric vehicles from the perspective of user experience and the evaluation method.

To sum up, the current research work mainly focuses on noise reduction by optimizing structure, noise reduction by using new materials, engine sound fitting, active sound system development, etc., and has achieved relatively fruitful research results. However, the development of ASGS in EV is not discussed in detail.

In this article, for an EV, comparing the sound difference between it and the same positioning fuel vehicle when driving, considering its power system, the dynamic characteristics and the output parameter information of the EV are quite different from those of the traditional fuel vehicle. In this article, researches are carried out on the interior sound design method, main control parameters selection and setting principle, active sound generation control system, test and evaluation of EVs, and the dynamic active control of the interior sound quality of EVs under acceleration condition is studied.

## 2. Sound analysis of in-vehicle engine orders

### 2.1. Comparative analysis of vehicles

**Fig 1:**
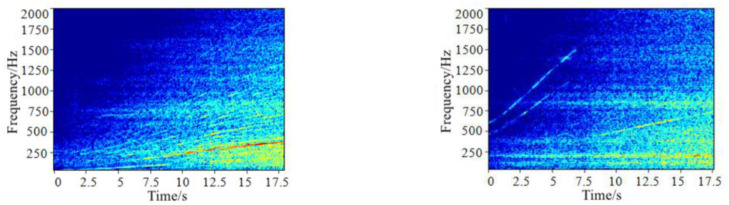
Spectrum comparison of vehicle sound during acceleration.

In the case of acceleration, the biggest difference between EV and FV is that EV does not contain the engine step sound mainly with medium and low frequency characteristics, while the motor order components and reducer gear engagement order components with high frequency characteristics appear, as shown in Fig 1.

According to the survey and analysis results of the internal sound development trend of EVs, engine sound is still one of the important development trends of EVs. Therefore, it is necessary to simulate the engine order sound in EVs.

### 2.2. In-vehicle engine order sound signal extraction

Some literatures [40, 41] divide engine sounds into engine order sounds and engine broadband sounds. It is quite similar to road noise. This research analyzes the engine order component sound.

A gasoline four-stroke engine’s ignition excitation frequency can be summed up as follows:

$$f_{e}=\frac{n_{e}}{2\times 60}\times N_{c}$$ (1)

Where *n*_*e*_ is the engine speed and *N*_*c*_ is the number of engine cylinders.

Within each complete engine speed, the ignition excitation of each cylinder is 0.5 times, that is, order 0.5 is the minimum interval of engine excitation order. Each engine stage corresponds to order 0.5 sound excitation. Its corresponding basic engine sound frequency *f*_1_ is as follows:

$$f_{1}=\frac{n_{e}}{2\times 60}$$ (2)

The 0.5 order component sound when the engine speed is *n*_*e*_ can be expressed by the sine wave signal as:

$$x_{1}(n_{e})=A_{1}\text{sin}(2\pi f_{1}+\varphi_{1})$$ (3)

Where *A*_1_ is the instantaneous amplitude of the fundamental frequency *f*_1_, *φ*_1_ is the instantaneous phase of the fundamental frequency *f*_1_.

When the engine speed is *n*_*e*_, the in-vehicle engine order sound can be expressed as follows:

$$X(n_{e})=\sum_{k=1}^{K}A_{k}\text{sin}(k\times \frac{\pi }{60}n_{e}+\varphi_{k})$$ (4)

Where *A*_*k*_ is the k-th engine order, that is, the instantaneous amplitude of the k/2-order component sound, *φ*_*k*_ is the k-th engine order, that is, the instantaneous phase value of the k/2-order component sound.

The continuous time signal of engine order sound is as follows:

$$X_{k}(t)=\sum_{k=1}^{K}A_{k}(t)\cdot \text{sin}[2\pi f_{k}t+\varphi_{k}(t)]$$ (5)

According to Formula (5), in order to ensure the fitting accuracy of engine order sound, it is necessary not only to accurately estimate the corresponding frequency of each engine order, but also to accurately calculate the amplitude and phase of each engine order sound component at any time.

### 2.3. The fourier transform of the engine order sound signal and the selection of the window function

For a continuous time-varying signal *x*(*t*), a time-varying window function *w*(*t*) with narrow time bandwidth is applied. In the process of Fourier transform, *w*(*t*) moves along the time axis, and its short-time Fourier transform (STFT) spectrum function is expressed as follows [42]:

$$STFT_{x}(t,f)=\int_{-\infty }^{+\infty }x(u)w^{*}(u-t)e^{-j2\pi fu}du$$ (6)

Fourier spectrum function *STFT*_*x*_ (*t*, *f*) is used for inverse transformation, which is also known as short-time Fourier synthesis, and the expression of the reconstructed time-varying signal is as follows:

$$x(t)=\int_{-\infty }^{+\infty }\int_{-\infty }^{+\infty }STFT_{x}(t,f)w(u-t)e^{j2\pi fu}dtdf$$ (7)

The mathematical expression of discrete STFT synthesis of discrete time signal *x*(*k*) is as follows:

$$x(k)=\sum_{n=-\infty }^{+\infty }\sum_{m=-\infty }^{+\infty }STFT_{x}(n,m)w(k△t-n△t)e^{j2\pi (m△f)}$$ (8)

Where *n* is the serial number of discrete time frames, *m* is the serial number of discrete frequency frames, Δ*t* is the sampling period of time variable, and Δ*f* is the sampling period of frequency variable.

If *X*_*n*_ (*m*) is the spectrum function of the discrete time signal in the complex form of frame *n* of *STFT*_*x*_ (*n*, *m*), where ${\tilde{a}}_{n}(m)$ and ${\tilde{b}}_{n}(m)$ are the real and imaginary parts of the function respectively, then the mathematical expressions of *X*_*n*_(*m*)’s amplitude ${\tilde{A}}_{n}(m)$ and phase ${\tilde{\varphi }}_{n}(m)$ are:

$${\tilde{A}}_{n}(m)=\sqrt[{2}]{{\tilde{a}}_{n}^{2}(m)+{\tilde{b}}_{n}^{2}(m)}$$ (9)

$${\tilde{\varphi }}_{n}(m)=\text{arctan}\left[{\tilde{b}}_{n}(m)/{\tilde{a}}_{n}(m)\right]$$ (10)

According to Formula (6), *STFT*_*x*_ (*t*, *f*) is actually the spectrum function of a narrowband time range of time signal *x*(*t*) at a certain time, and the accuracy of time resolution Δ*t* depends on the time width of window function *w*(*t*). The smaller the time, the more accurately *STFT*_*x*_ (*t*, *f*) can describe the spectrum characteristics at this time, that is, the higher the time resolution. On the other hand, the frequency resolution Δ*f* depends on the width within the frequency range of the window function. The narrower the frequency, the higher the frequency resolution.

However, according to Heisenberg uncertainty criterion principle of STFT analysis window function, Δ*t* and Δ*f* has the following relation: Δ*t* × Δ*f* × ≥ 1/4*π* [43]. It is known that it is impossible to obtain high-precision time resolution and frequency resolution at the same time through STFT, so the selection window function is very important.

For this article, the ideal window function can obtain better frequency resolution in low frequency band and better time resolution in high frequency band, and requires adjustable main lobe width and low amplitude side lobe peak value to reduce frequency leakage.

Kaiser window has high flexibility and can be adjusted by controlling shape parameters *β* and length *N*. It is widely used in incident signal analysis in various engineering fields.

For the in-vehicle engine order sound, according to Formula (2), the calculation formula of frequency difference Δ*f* between two adjacent engines is as follows:

$$\Delta f=\frac{k\times n_{\text{e}}}{2\times 60}-\frac{(k-1)\times n_{\text{e}}}{2\times 60}=\frac{n_{\text{e}}}{120}$$ (11)

When the engine speed rises from 750 r/min to 6000 r/min, the frequency difference Δ*f* between the two adjacent engines rises from 6.25 Hz to 50 Hz, the engine order sound fundamental frequency *f*_1_ = Δ*f*, and when the main lobe width *B* ≤ *f*_1_ of the Kaiser window function, all engines can be clearly identified by the sound frequency.

## 3. Sound simulation

### 3.1. Engine order sound test

In order to verify the feasibility of the above technology, it is necessary to accurately obtain the original time domain data of the vehicle engine order sound, and also to obtain the synchronous data changes within the engine speed time domain range, used for the accurate estimation of the engine order sound base frequency and each frequency in the process of STFT data. Then obtain the amplitude and phase of each engine order frequency component. The following introduces the test process of the engine order sound in FV:

Step1. Prepare for the test. The type SCR205 multi-channel data acquisition equipment produced by Siemens was used for testing. The 4189-A-021 microphone manufactured by Danish B&K company was placed in the driver’s right ear according to the layout method of Chinese national standard GB/T 18697–2002 Acoustic Vehicle Interior Noise Measurement method. The vertical distance from the seat surface to the backrest surface is (0.70 ± 0.05) m above the intersection line, and the horizontal coordinate is (0.20 ± 0.02) m from the seat center surface (The tests carried out in this paper have obtained the written consent of the driver.). In addition, a speed sensor is arranged near the engine camshaft to detect and collect the change of engine speed.

Step2. Vehicle sound and engine speed signal acquisition. On the horizontal and smooth asphalt road in the test site, set the transmission gear to the third gear and slowly press down the accelerator pedal to accelerate the engine speed uniformly from the lowest engine speed in this gear to the rated engine speed. The whole acceleration time is no less than 20 s. The sampling frequency of vehicle sound signal and engine speed signal is set to 51.2 kHz.

**Fig 2:**
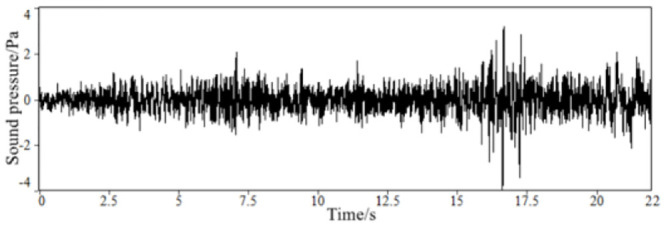
The time-domain signal of the sound inside FV when accelerating.

**Fig 3:**
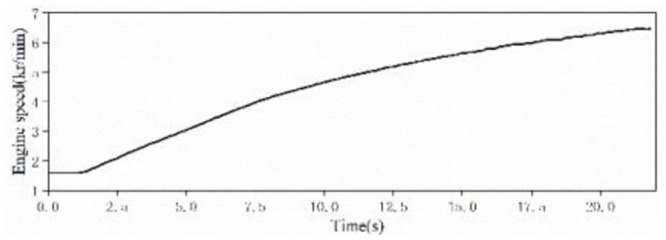
The time-domain signal of engine speed of a FV when accelerating.

**Fig 4:**
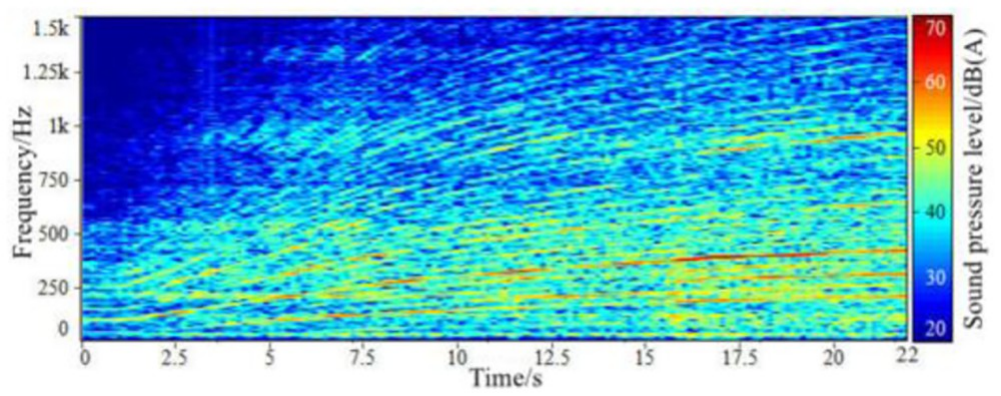
The frequency spectrum of sound inside a FV when accelerating.

Through the above steps, the time-domain signals of the sound inside the accelerated driving vehicle and engine speed are obtained, as shown in Figs 2 and 3. With the help of the third-party software specially used for acoustic analysis, the FFT spectrum analysis of the sound in the accelerated vehicle is carried out. The results of the spectrum analysis of the vehicle are shown in Fig 4.

### 3.2. Sound synthesis and validation

**Fig 5:**
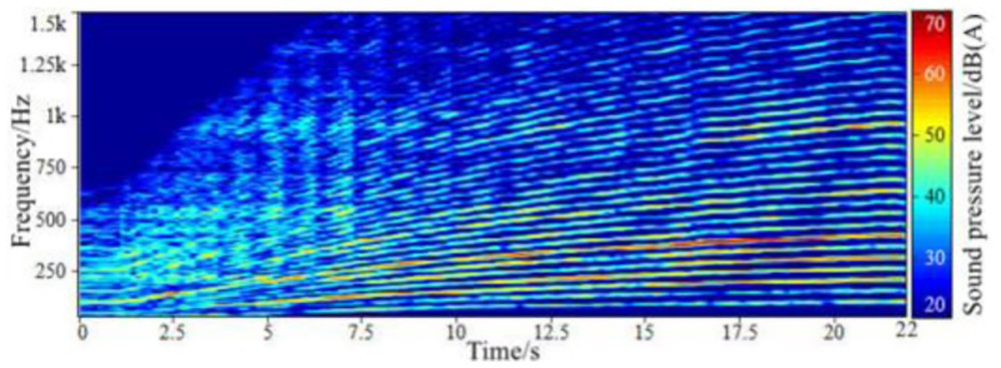
The frequency spectrum of synthetic sound.

The analysis window is Kaiser window, the value of *β* is 5.8, the window length is 1280, the FFT length is 4096, the overlap rate is 50%, and the time frame length is 0.05 s. According to the in-car sound spectrum in Fig 5, when the effective number of engine order components K is 40, that is, the component sound of order 20 of engine speed is sufficient to characterize the characteristics of engine order sound. Therefore, the engine speed signal collected synchronically with the sound signal is taken as the input. According to Formula (1), the first 20 orders of engine harmonic frequencies in the process of slow acceleration of engine speed were estimated, and the amplitude and phase of 40 engine harmonic frequencies in each time frame were calculated according to Formula (9) and (10).

**Fig 6:**
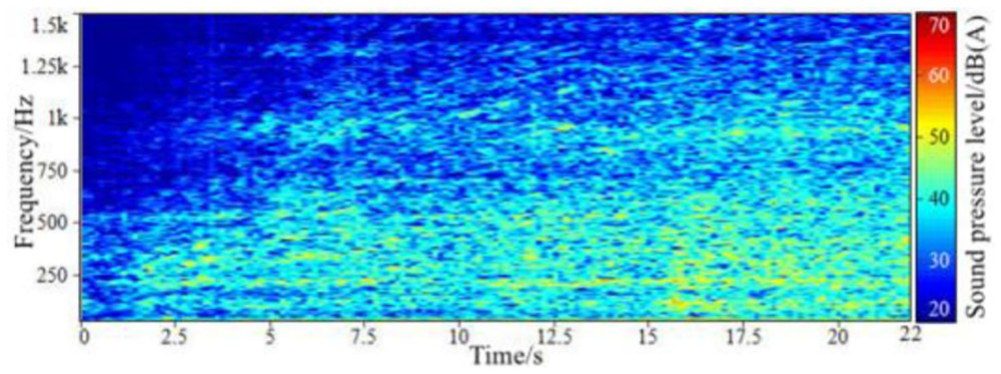
The spectrum of the background sound in-vehicle when accelerating.

**Fig 7:**
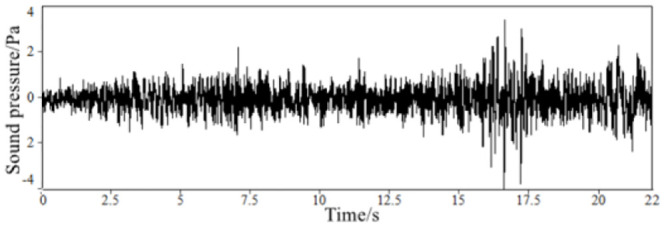
The time-domain signal of synthetic in-vehicle sound during acceleration.

**Fig 8:**
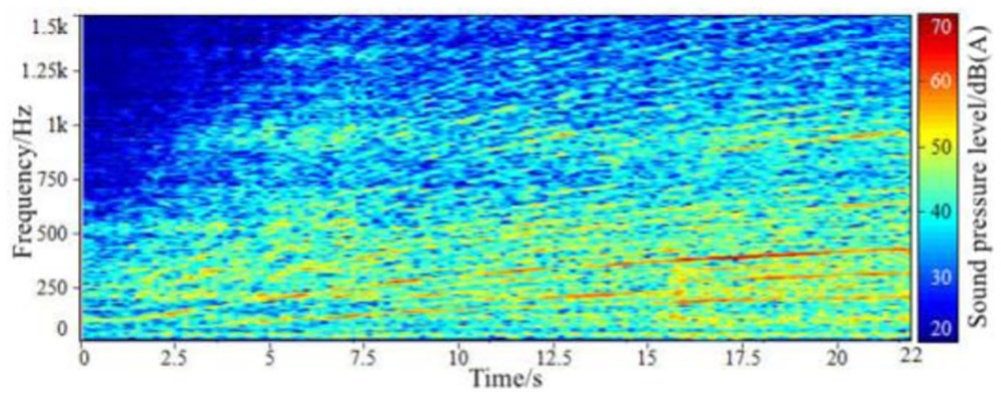
The spectrum of the synthetic sound in-vehicle when accelerating.

Finally, by short-time Fourier comprehensive, complete the engine order ingredients sound simulation, at the same time using third-party acoustic processing software LEA will sound engine order components and other background sounds, the vehicle will get the engine integrated of the STFT order ingredients sound mixing with other background sounds inside the vehicle, get a synthetic speed up the vehicle. The synthesized sound spectrum of engine order components in the vehicle is shown in Fig 5, and the background sound spectrum of the accelerated vehicle separated by LEA software is shown in Fig 6. Therefore, the time domain signal and FFT spectrum of the synthesized sound in the accelerated vehicle after the mixing of the two are shown in Figs 7 and 8.

Through comparing the sound spectrum of the synthesized accelerated vehicle with that of the tested accelerated vehicle, it can be seen that: After the short-time Fourier transform and synthesis technology, the order component sound of the in-vehicle engine is obtained. The main energy area and the change law of the main order component amplitude are not much different from the original input signal, and there are slightly different in the low-speed region and the high-order sub-region, and the amplitude changes of some order components have a certain degree of discontinuity.

### 3.3. Subjective evaluation

**Fig 9:**
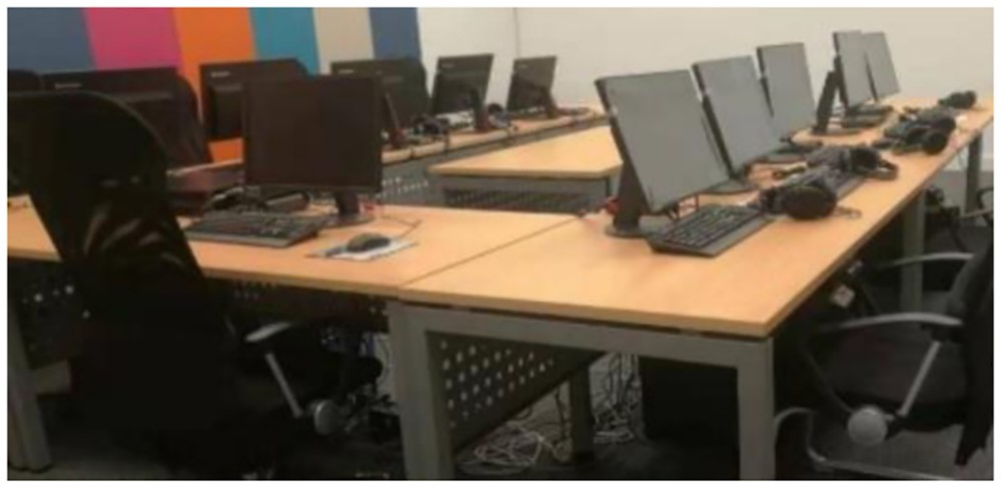
Sound playback and subjective evaluation system based on HEAD Acoustics.

In Fig 9, the subjective evaluation test was conducted by experienced NVH subjective evaluators who scored the subjective evaluation only based on the sound sample. In order to improve the work efficiency, this article will use the acquisition voice and playback mode, are able to carry out subjective evaluation is more effective and flexible, at the same time this way support "blind type" subjective evaluation, the evaluators in the case of not know vehicle models to evaluate, can avoid the type of vehicle effect. In the audio playback and subjective evaluation system, the evaluator can perform multiple playback auditions by himself. The results showed that there was little difference in overall subjective auditory perception between the two. The synthetic acceleration vehicle sound is slightly different from the original sound signal in the aspect of sound realism, mainly due to the engine order component composition error in the high frequency region.

Information about NVH engineers is shown in Table 1. The grade scoring method is chosen as the subjective evaluation method. In this method, an attribute of sound quality is taken as an evaluation index, and the subjective feelings caused by it are divided into ten grades. Each grade corresponds to different scores. In the experiment, the evaluators give corresponding scores according to their understanding of this attribute of sound and their own subjective feelings. The sound comfort quality and dynamic quality were used as evaluation indexes. The subjective perception intensity is shown in Table 2.

**Table 1:** Subjective evaluation team personnel information.

Evaluator	Gender	Age	Professional	Working years
A	male	35	NVH subjective evaluation	11
B	male	30	NVH subjective evaluation	6
C	Female	34	Sound quality research	9
D	male	32	NVH subjective evaluation	7
E	male	34	Sound quality research	10
F	male	40	Performance development	14
G	male	38	Vehicle NVH performance development	11
H	male	35	NVH subjective evaluation	9

**Table 2:** Evaluation grades.

Score	Subjective perception	Satisfaction
1	Unacceptable	No quality
2		
3	Very poor	
4		
5	Improvement needed	Improvement is desired
6	Acceptable	Quality is generally satisfactory
7	Good	
8	Pretty good	Obvious quality
9	Very good	Very strong quality
10	Great	

**Fig 10:**
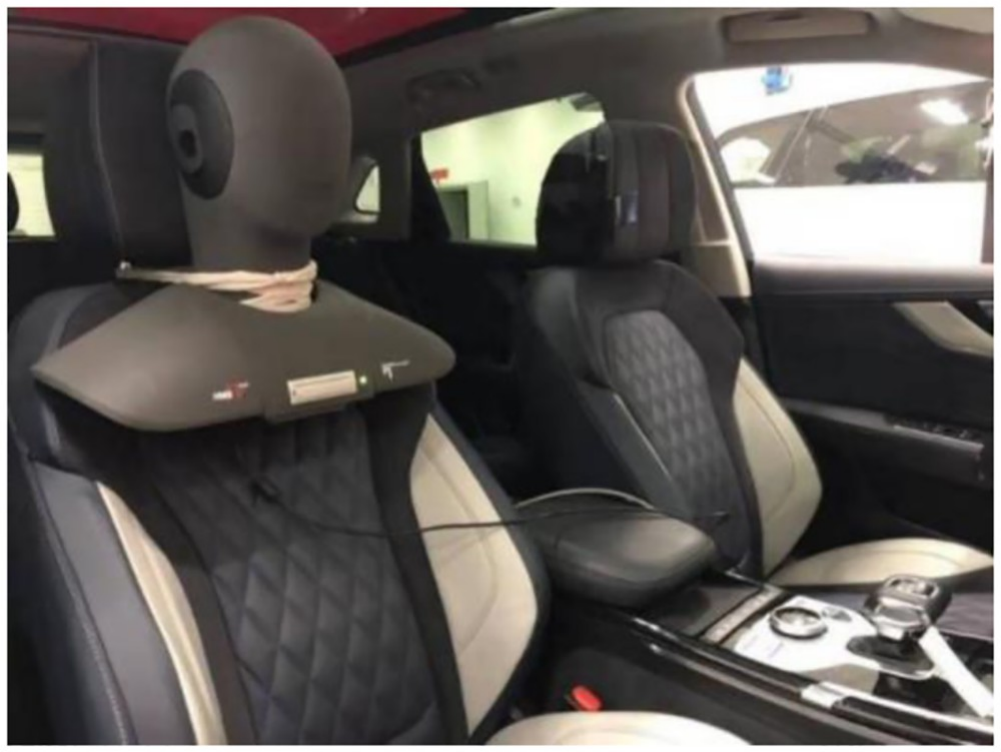
Layout of sound measuring points in HEAD Acoustics artificial head.

The HEAD Acoustics artificial head is used to collect the interior sound of the sample vehicle, as shown in Fig 10.

We selected 12 FVs and 2 EVs for sound testing. Tests on proving grounds and smooth roads are conducted for the in-vehicle sound tests. The sample vehicle information is shown in Table 3.

**Table 3:** Basic information of test sample vehicle.

Number	Vehicle type	Engine	Capacity/L
1	Class C SUV	V8	5.7
2	Class C SUV	V8	5.0
3	Class B sedan	I4	2.0
4	Class A MPV	I4	1.5
5	Class C sedan	V6	3.0
6	Class C sedan	I4	2.0
7	Class A electric SUV	——	——
8	Class C sedan	I4	2.0
9	Class B sedan	I4	2.0
10	Class B SUV	I4	2.0
11	Class C sedan	I4	2.0
12	Class B SUV	V6	3.0
13	Class A coupe	I4	2.0
14	Class A EV	——	——

**Fig 11:**
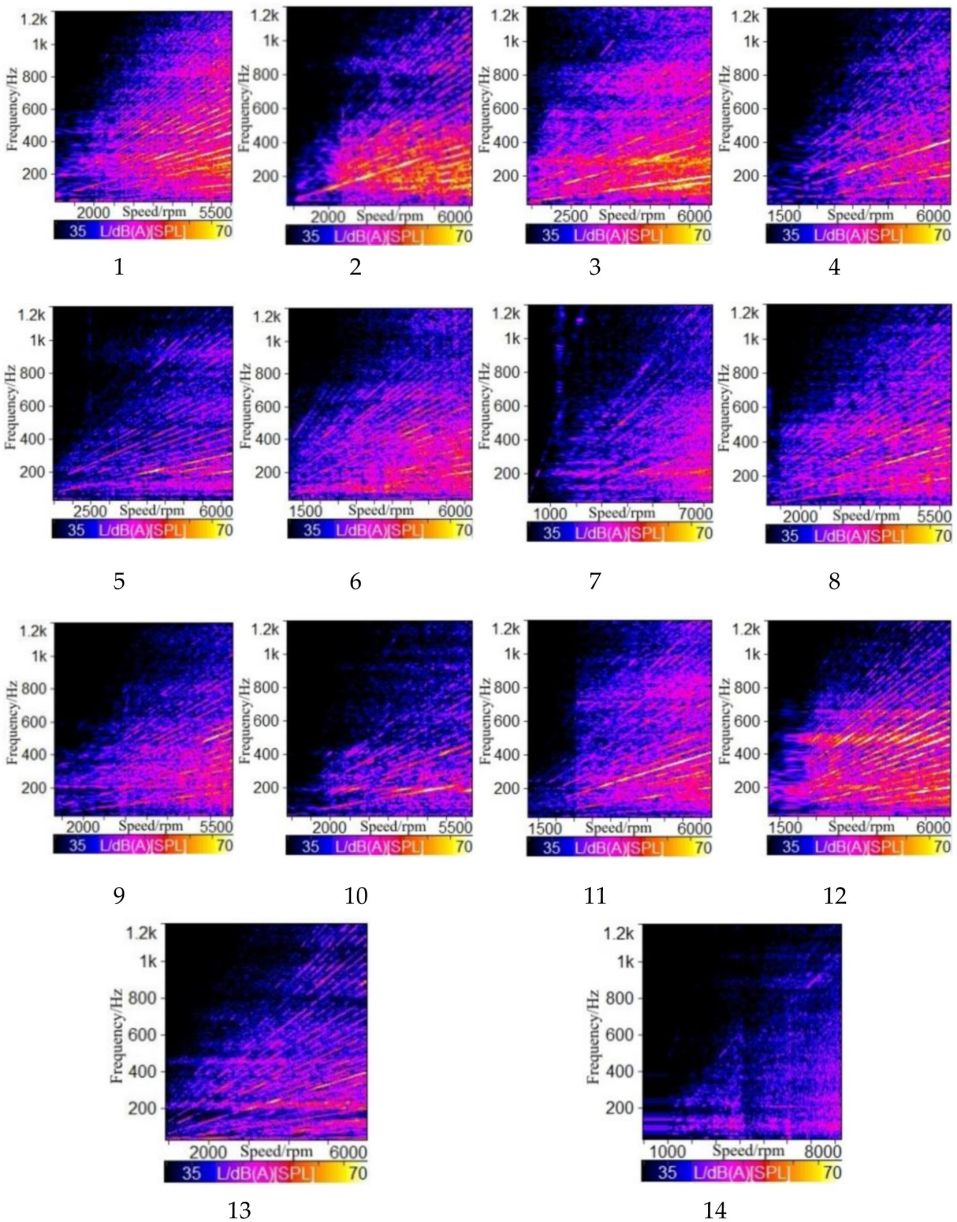
FFT acoustic spectrum in each sample vehicle.

**Fig 12:**
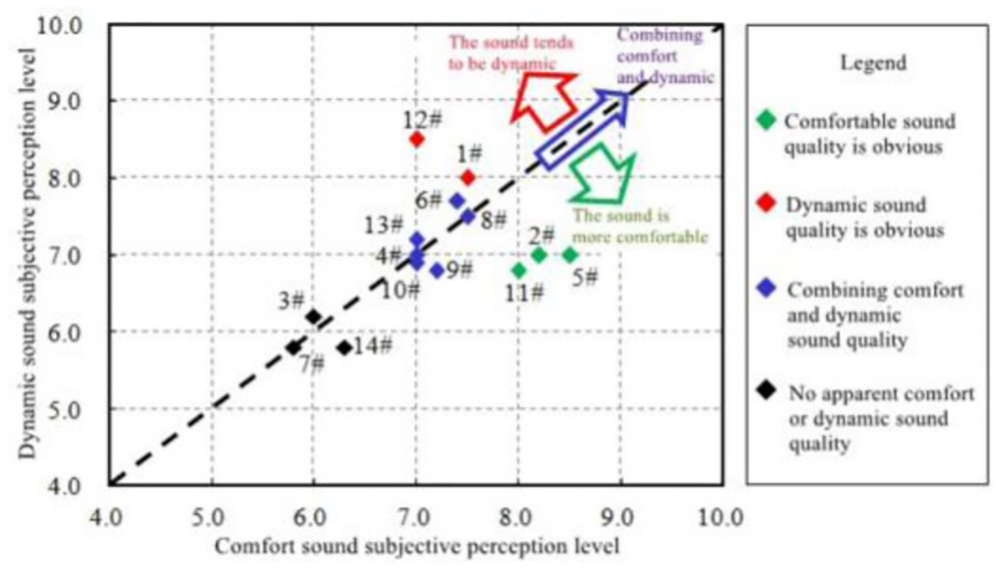
Subjective evaluation of sound quality in the sample vehicle.

FFT spectrum analysis was performed on 14 sample vehicles, as shown in Fig 11. Fig 12 shows the distribution of subjective evaluation results.

In general, the STFT based on Kaiser window function and the integrated technology can meet the requirements of the fitting accuracy of the in-vehicle engine sound, and can be used to simulate the in-vehicle ASGS of EVs.

## 4. Active sound generation system of EV

### 4.1. Design and analysis of EV sound

The research object of this article is an A-class pure electric SUV, and the following conceptual design goals for the interior sound of an electric vehicle are formulated: the interior sound of the SUV should have both the sound characteristics of comfort and power.

However, comfort and power are often contradictory sound quality requirements. The former requires a low-amplitude and smoothly changing engine sound, while the latter requires a higher-amplitude engine sound with obvious dynamic changes.

In order to realize the design and analysis of the sound of EVs, it is necessary to understand the influence of engine order composition, energy distribution in frequency domain and local enhancement of sound amplitude in speed domain on sound quality.

#### 4.1.1. Influence of engine order composition and energy distribution

According to the previous research of our team, the following conclusions can be drawn that the main characteristics of vehicle sound in accelerated driving with typical comfort quality are as follows: low amplitude of vehicle background sound which can ensure high speech articulations. And it has rich engine order components, and the engine order components in the range below 400 Hz are clearer than those in the range above 400 Hz. The main characteristics of vehicle sound in accelerated driving with typical power sense quality are as follows: there are rich engine order components during driving, the ratio of engine order component sound to background sound is slightly larger, and the engine order component above 400 Hz is clearer than the engine order component below 400 Hz.

To sum up, both the comfortable sound quality and the power sound quality require relatively rich engine order components, and the prominent main order components of the engine will enhance the comfortable sound quality. In addition, by balancing the energy of the engine order components below 400 Hz and the energy of the engine order components above 400 Hz, it is possible to achieve appropriate adjustment of the comfort and power feeling of the sound in the acceleration driving vehicle.

#### 4.1.2. Determination of local enhancement region

**Fig 13:**
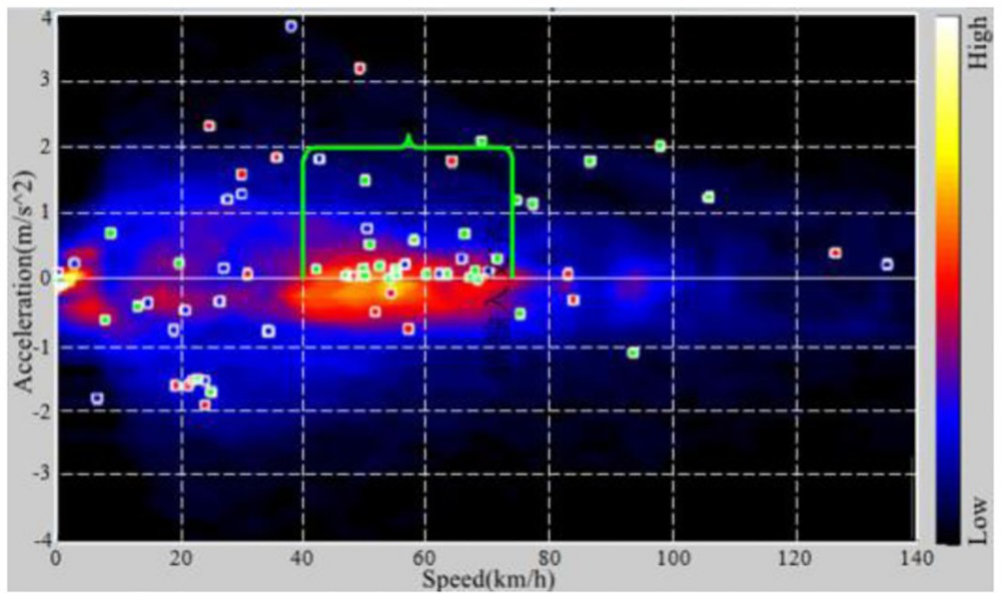
Dynamic driving behavior distribution map.

Literature [44] carried out a study on dynamic driving behavior of drivers with a certain number of samples for an EV, statistically analyzed the probability of acceleration, uniform speed and deceleration of drivers within the speed range of 0–140 km/h, and drew a color cloud map of probability distribution of dynamic driving behavior, as shown in Fig 13. The color in the figure indicates the probability of occurrence of the corresponding driving condition of the driving speed and acceleration, and the brighter the color of the area indicates the higher the probability of the occurrence of the corresponding driving condition in that area.

According to Fig 13, the vehicle speed range in which the driver is most likely to perform acceleration, constant speed and deceleration driving operations can be summarized, as shown in Table 4. Among them, 40–75 km/h is the speed range in which the driver has the highest probability of accelerating and driving. It is the preferred speed range that needs to improve the sound quality of the vehicle’s dynamic sense through the active sound system, which can appropriately increase the engine sound in this speed range.

**Table 4:** The most likely speed range under each driving condition.

Driving condition	Most likely speed range (sorted by likelihood)
Accelerated driving	40-75km/h>0–6 km/h >15–20 km/h
Reduce speed	35-75km/h>0-10km/h>15-30km/h
Uniform driving speed	40-80km/h>0-6km/h

In order to facilitate the control of the sound frequency of the active sound system of EVs, the variable of virtual engine speed is set between the vehicle speed and the sound frequency, and the virtual engine speed is calculated from the speed of the vehicle, and the system synthesizes the corresponding frequency according to the virtual engine speed.

**Fig 14:**
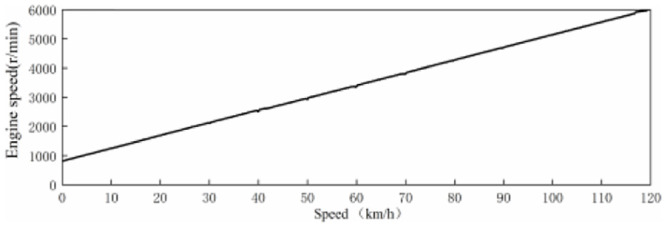
Relationship curve between virtual engine speed and vehicle speed.

When the speed reaches 120 km/h or above, the driver’s driving behavior is mainly slow acceleration, uniform speed or deceleration [44], and the vehicle sound is mainly wind noise, engine sound provides little sound perception for the driver under the driving condition above 120 km/h. Meanwhile, considering that the maximum speed limit on Chinese road is 120 km/h, therefore, the speed range of active sound in EV is defined as 0–120 km/h, that is, the speed of 0 km/h corresponds to the virtual engine speed of 750 r/min, and 120 km/h corresponds to the virtual engine speed of 6000 r/min. Meanwhile, the virtual engine speed shows a linear change trend with the speed. Fig 14 shows the relation curve between virtual engine speed and speed, and its calculation formula is as follows:

$$RPM_{VE}=43.75\times V+750$$ (12)

Where *RPM*_*VE*_ is the virtual engine speed, *V* is the speed.

Among them, the virtual engine speed range corresponding to the 40–75 km/h speed range is roughly 2500–4000 r/min.

### 4.2. Sound design of active generation sound system when accelerating

#### 4.2.1. Set the target of vehicle sound value when accelerating

According to the above sound design principles in an accelerating vehicle, the following specific sound design requirements are determined from the three dimensions of engine order composition, frequency domain energy distribution, and sound amplitude enhancement in a typical vehicle speed range:

①In terms of engine order structure, 4th engine order 4 should be the main order, supplemented by integer order and half-order of other engines. Meanwhile, the amplitude of engine main order sound should be moderate, and engine order components should cover the range of 20–1200 Hz.

②In terms of energy distribution in the frequency domain, the sound amplitude of the engine order components below 400 Hz should be greater than that of the engine order components above 400 Hz.

③In terms of sound amplitude enhancement in typical vehicle speed range, it is necessary to appropriately enhance the sound amplitude of engine order components in 2500–4000 r/min virtual engine speed range.

In addition to the above sound design requirements, the change of engine order component sound amplitude should conform to the general change law, for example, the overall change of engine order sound amplitude should show a trend of gradually increasing with the engine speed.

**Fig 15:**
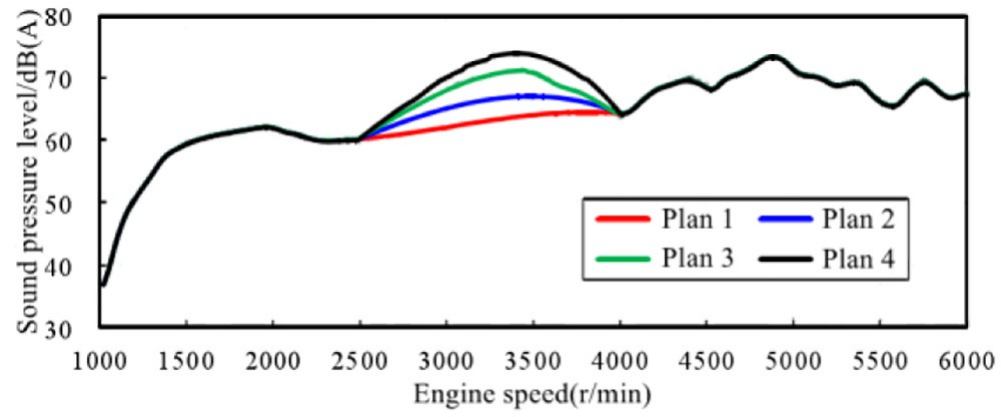
Alternatives for in-vehicle sound design while accelerating.

**Fig 16:**
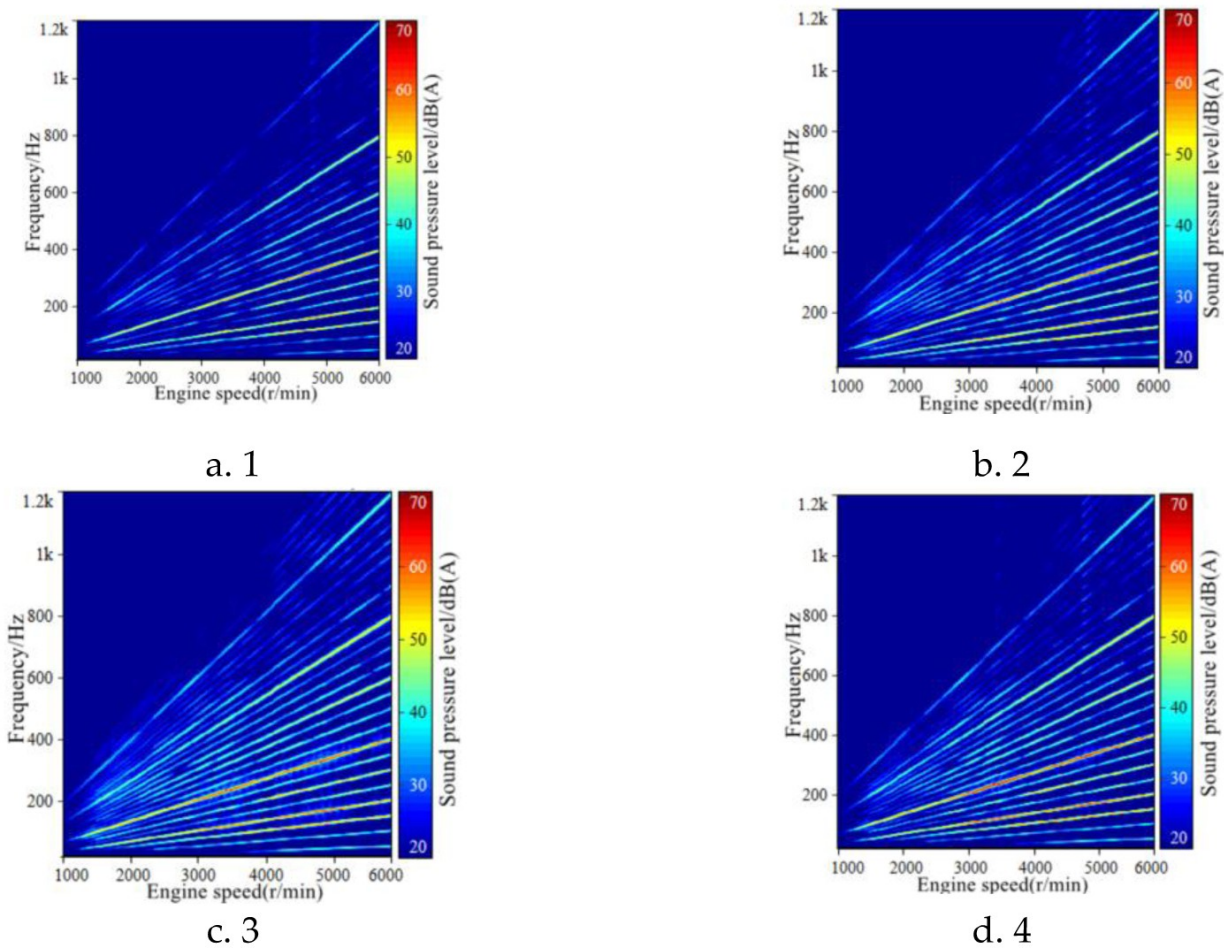
The FFT spectrum of the four schemes with engine speed.

Engine order component sound was constructed in the Active Sound Design module of Genesis’ LEA acoustic processing software. At the same time, in terms of the requirement of sound amplitude enhancement in typical speed range, different sound amplitude gain sound schemes are formulated for 2500–4000 r/min virtual engine speed range, and finally four alternative schemes are obtained. Fig 15 shows the variation curves of total sound value of the four schemes with engine speed, and Fig 16 shows the FFT spectrum of the four schemes with engine speed.

For the four schemes, the subjective evaluation was carried out according to the same method in 2.2. The results show that the acceleration sound of scheme 1 and scheme 2 is more comfortable, and the power quality is relatively weak. Scheme 4 has obvious power sense, but the comfort quality is weak. In scheme 3, the sound in the accelerated driving vehicle can have both comfort and power. Therefore, this sound design scheme is selected for the active sound generation control system in the vehicle of EVs.

#### 4.2.2. Set the target of engine order sound value when accelerating

In 3.2.1, the main engine order composition, frequency domain energy distribution and relative amplitude between orders are clarified, on this basis, the real vehicle driving conditions also need reasonable objective parameters, considering the real vehicle ASGS under the condition of the voice with the original vehicle engine order times background sound relations of relative amplitude. Therefore, it is necessary to refer to a traditional FV with the same positioning and target sales group as the EV to analyze the correlation between the amplitude of the engine’s order sound in the vehicle and other background sounds in the vehicle, so as to set the sound amplitude of the active sound generation control system.

The team has verified in the early stage that the sound quality characteristics of a coupe-type traditional FV are consistent with the sound concept design goals of this article. Therefore, this section will take the traditional FV’s in-vehicle sound as the research object, and analyze the relationship between the in-vehicle engine order sound amplitude and the background sound amplitude when the car is in the 3rd gear and 100% accelerator pedal opening.

**Fig 17:**
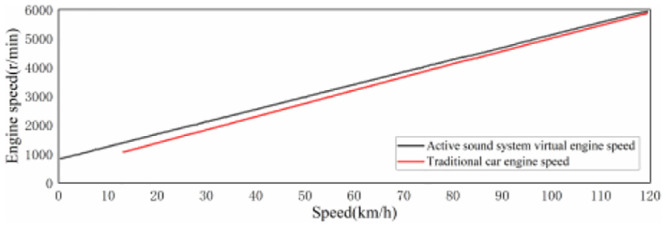
Comparison between traditional engine speed of FV and active sound system virtual engine speed.

In the process of accelerating the car in 3rd gear, the engine speed is accelerated from 1000 r/min to 6000 r/min, and the vehicle speed is accelerated from 13 km/h to 121 km/h. Calculate the virtual engine speed and vehicle speed of the ASGS according to formula (12). In Fig 17, it can be seen that there is little difference between the two, indicating that the total value of sound in the acceleration driving condition with 100% accelerator pedal opening of the ASGS can be set according to the interior sound of the traditional FV in the same driving condition.

**Fig 18:**
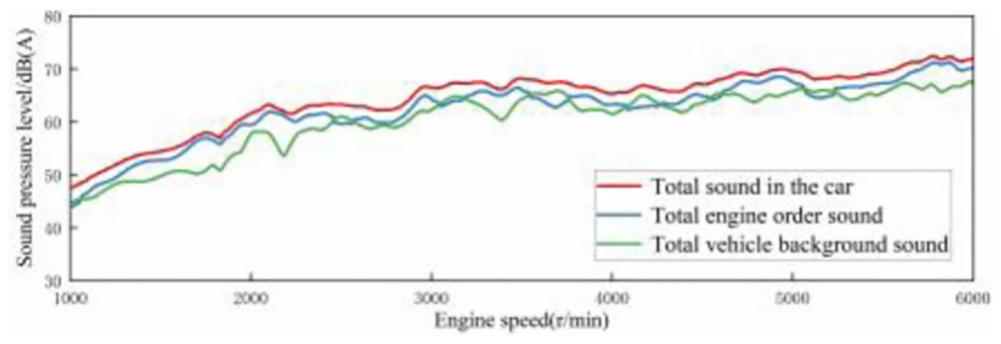
Sound condition of a traditional coupe when accelerating at 100% accelerator pedal opening.

In this article, LEA, the acoustic processing software of French Genesis company, was used to extract the engine order components from the sound spectrum of the accelerated vehicle. The engine order sound can be separated from the background sound, as shown in Fig 18.

**Fig 19:**
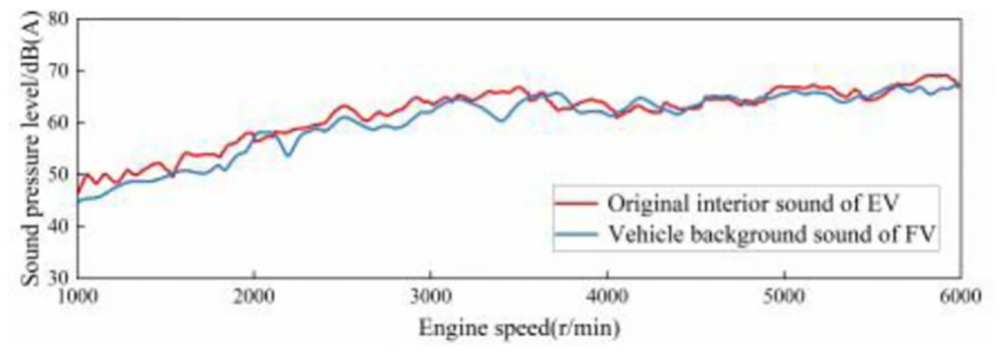
Comparison of total value curve of background sound inside EV and FV.

**Fig 20:**
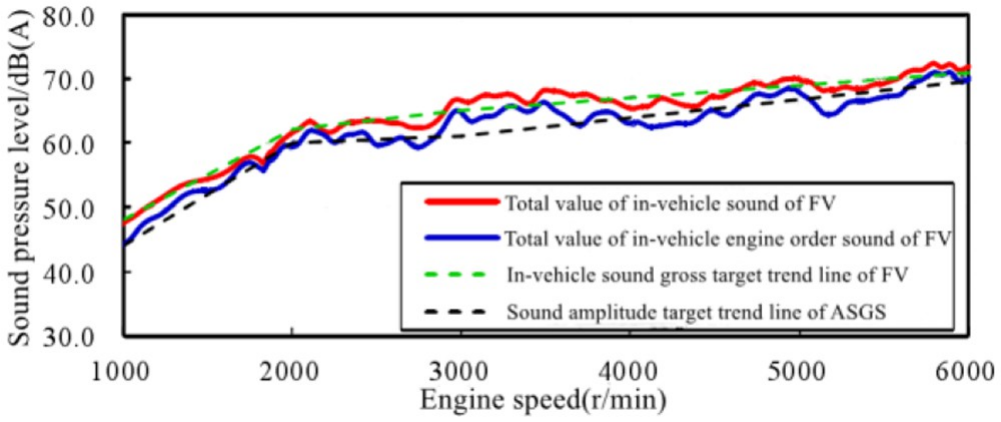
Target trend line of sound amplitude of ASGS at 100% accelerator pedal opening condition.

The EV studied in this article is also accelerated at 100% accelerator pedal opening, and the interior sound is analyzed, and the total value of interior background sound is compared with that of traditional FV which studied in this article, as shown in Fig 19. It can be seen that the variation trend of the two is basically the same in the whole speed range. Therefore, the sound amplitude target trend line of the ASGS as shown in Fig 20 and the overall target trend line of the sound inside the selected electric vehicle are formulated according to the changes of the sound inside the traditional FV in Fig 18.

**Fig 21:**
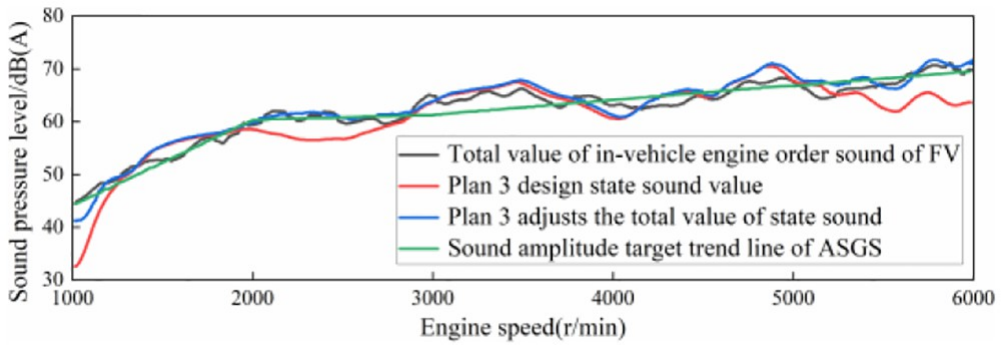
Objective curve of total sound value changing with engine speed at 100% accelerator pedal opening in scheme 3.

According to the target trend line of sound amplitude of the ASGS, adjustment was made to scheme 3, in which 2500–4000 r/min sound amplitude enhancement area remained unchanged, and the curve of sound amplitude of the system at 100% accelerator pedal opening was obtained, as shown in Fig 21. It can be seen that the adjusted total sound curve of the system is not only consistent with the trend of the total sound of the engine in the whole speed region, but also retains the sound amplitude enhancement area of 2500–4000 r/min.

### 4.3. Control parameter selection of ASGS

The change of the speed can reflect the driving state of the vehicle, and the system needs to determine the sound frequency according to the change of the speed. The parameters of the ASGS of the EV studied in this article are adjusted by using the sound characteristics of the matched traditional FV.

The variable of virtual engine speed has been set, the virtual engine speed is calculated by the speed of vehicle, and the ASGS synthesizes the engine sound of corresponding frequency according to the virtual engine speed.

**Fig 22:**
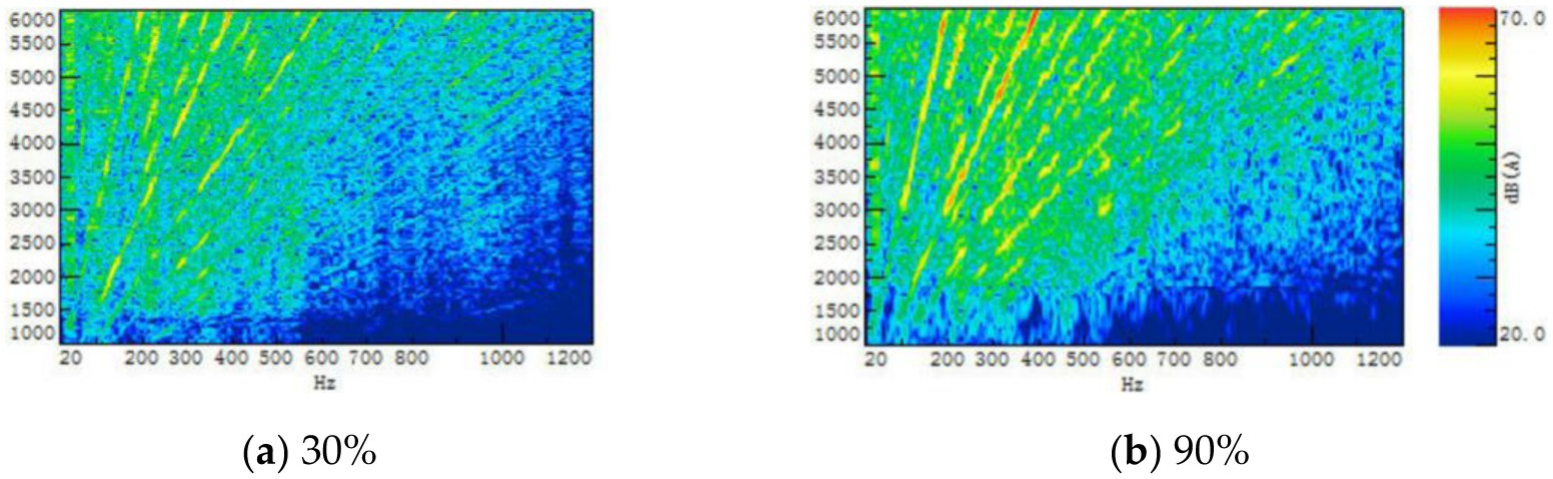
Comparison of sound spectrum in vehicle when accelerating under different accelerator pedal opening.

Another important parameter of the ASGS is the change of pedal opening, which can well reflect the dynamic characteristics of the vehicle. The power output of the powertrain is different with the opening of the pedal, leading to the amplitude of the sound in the vehicle will be different. Fig 22 is part of the comparison of the spectrum of sound inside a traditional FV when the engine speed is accelerated from 1000 r/min to 6000 r/min at different accelerator pedal openings. Taking 30% and 90% accelerator pedal opening as an example, it is obvious that the engine order sound in the vehicle accelerating at 90% opening is greater than that at 30% opening. Therefore, the ASGS of EV can realize sound amplitude control through the recognition of the opening of the accelerator pedal.

#### 4.3.1. Dynamic sound characteristics of FV

**Fig 23:**
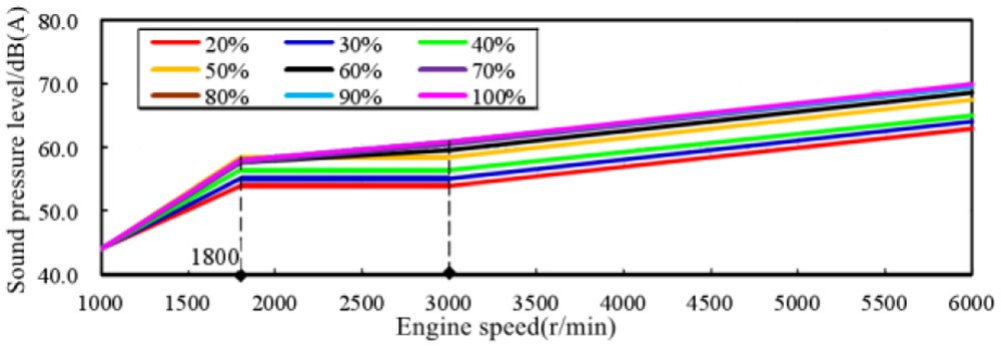
Variation trend of engine order sound amplitude with different pedal opening.

The changes of in-vehicle engine order sound amplitude with engine speed trend under different pedal opening conditions are summarized, as shown in Fig 23.

It can be seen from the figure that the overall trend change of the in-vehicle engine order sound amplitude can be divided into three parts:

1. The speed range of 1000–1800 r/min is the initial rapid change area of the amplitude, and the sound amplitude increases rapidly to the corresponding position from the same position in each working condition.
2. The speed range of 1800–3000 r/min is a smooth transition zone of amplitude, and the sound amplitude of each working condition remains stable or increases slowly.
3. The speed range of 3000–6000 r/min is a stable growth area, and the sound amplitude of each working condition increases steadily with the engine speed.

The engine speed range of 3000–6000 r/min was selected as the first choice for correlation analysis between sound amplitude and engine output power. The main reason is that this range has the following three characteristics:

1. the characteristics of engine sound order are most obvious.
2. The output power of the engine gradually increases and reaches the peak.
3. The amplitude trend of the engine order sound is relatively consistent.

**Fig 24:**
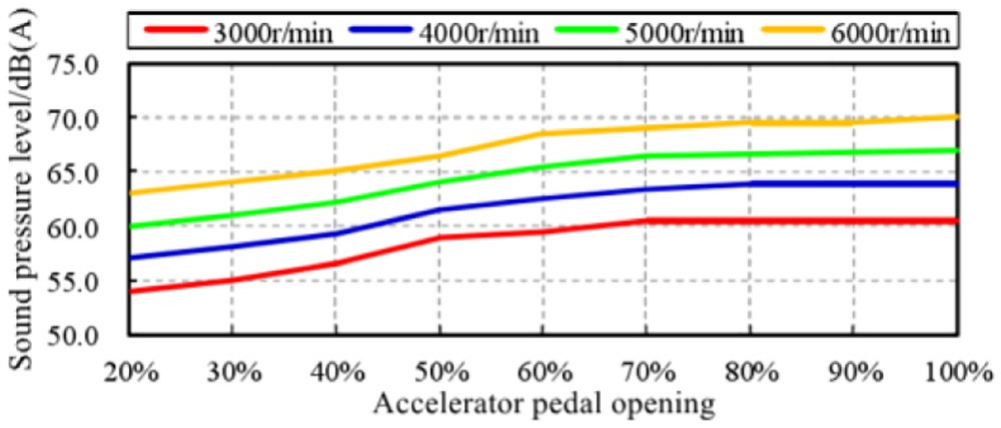
The amplitude trend of engine order sound changes with the opening of accelerator pedal.

**Fig 25:**
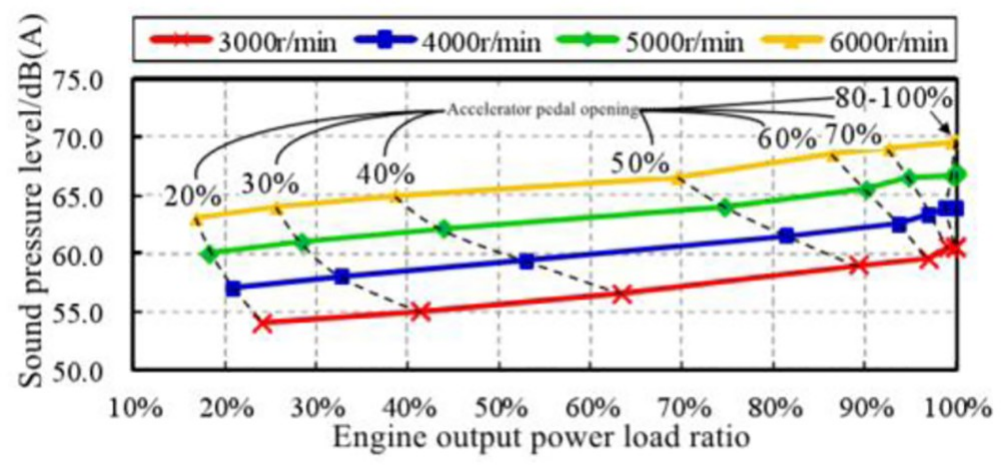
The amplitude trend of in-vehicle engine order sound changes with engine output power load ratio.

Extract the sound amplitudes of engine speeds of 3000 r/min, 4000 r/min, 5000 r/min, 6000 r/min at each accelerator pedal opening from Fig 23, and analyze the correlation with the pedal opening and engine output power. In order to facilitate the correlation analysis between sound amplitude variation trend and engine output power, the concept of engine output power load ratio is introduced here. It is the ratio of engine output power at a certain accelerator pedal opening to engine output power at 100% accelerator pedal opening at the same engine speed. The trend of the in-vehicle engine order sound amplitude trend with the pedal opening is shown in Fig 24, and the curve with the engine output power load ratio is shown in Fig 25.

**Fig 26:**
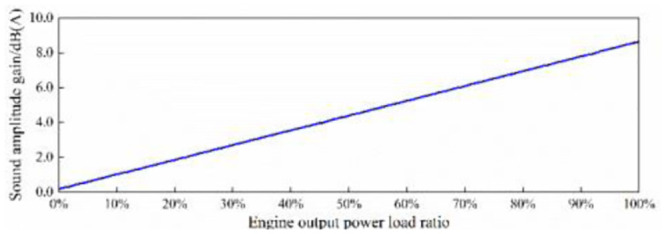
Curve of in-vehicle engine order sound amplitude gain with engine output power load ratio.

At any engine speed in the range of 3000–6000 r/min, the amplitude trend of in-vehicle engine order sound basically shows a linear relationship with the load ratio of the output power of the engine. That is, when the engine output power load ratio *η*_*p*_ increases by Δ*η*_*p*_, the engine’s order sound amplitude trend increases by Δ*L*_*A*_, and Δ*L*_*A*_ = α·Δ*η*_*p*_, and the sound amplitude gain coefficient *α* is constant. According to Fig 24, the sound amplitude gain coefficient *α* at each engine speed can be calculated, and the mean value of the sound amplitude gain coefficient *α*_*AVE*_ = 0.85 ⋅ [*dB*(*A*)/100%] can be further calculated, as shown in Table 5. By applying it to the total power output load ratio of the engine, the amplitude gain of in-vehicle engine order sound is plotted with the change of engine output power load ratio, as shown in Fig 26.

**Table 5:** Sound amplitude gain coefficient calculation.

Engine speed(r/min)	*α*[*dB*(*A*)/100%]	*α*_*AVE*_[*dB*(*A*)/100%]
3000	8.1	8.5
4000	8.5	
5000	8.8	
6000	8.8	

#### 4.3.2. Parameter setting principle

Because the structure and working principle of engine and motor are very different, their power output characteristics are also very different. Therefore, it is necessary to study the variation rule of the output power of the driving motor during the acceleration of EVs, and compare it with the variation rule of the engine output power, so as to provide a basis for the formulation of the active sound control strategy considering the dynamic change characteristics of the dynamic induction.

For the EV used in this article, vehicle parameters such as vehicle sound, speed, motor speed, motor torque and accelerator pedal openness were tested under different accelerator pedal openness. The preparation of the hardware and software used for the test, and the arrangement of the sound sensors in the EV are similar to those in traditional FV. The test conditions are slightly different: the transmission gear is placed in D, and the driver quickly presses the accelerator pedal and keeps it open at 20%. The motor speed accelerates from 0 r/min to 6000 r/min, and simultaneously collects and records the vehicle sound signal and the speed, motor speed, motor torque, accelerator pedal opening and other signals in CAN information. Repeat the above test process at 30%, 40%, 50%, 60%, 70%, 80%, 90% and 100% accelerator pedal opening respectively.

**Fig 27:**
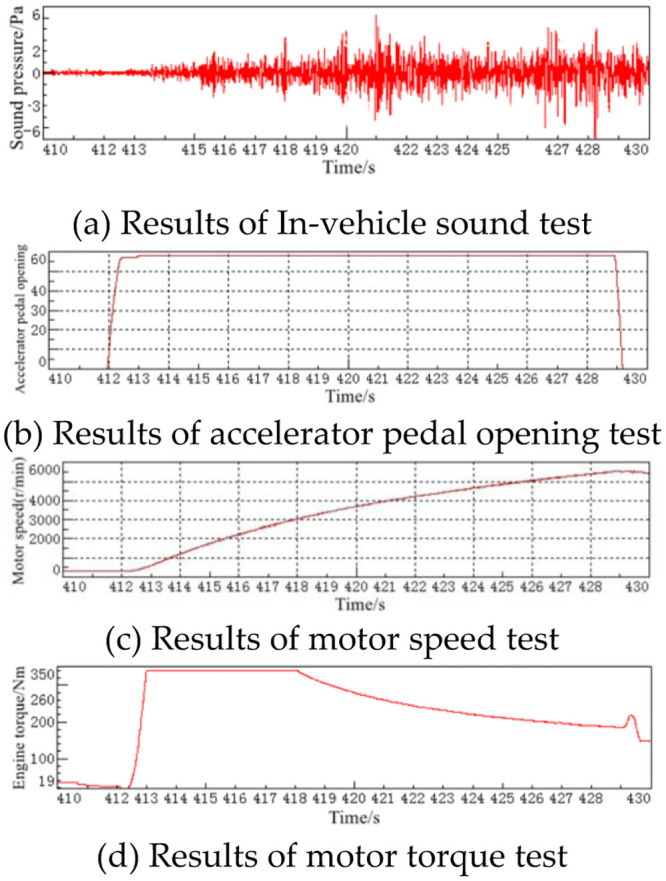
Each test signal curve at 60% accelerator pedal opening.

The test results of the vehicle at 60% acceleration pedal opening are shown in Fig 27, and other conditions are not described here.

**Fig 28:**
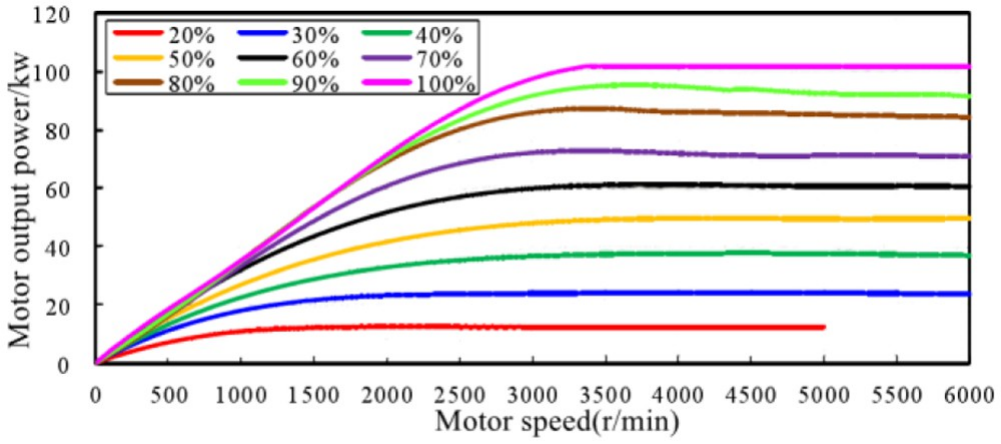
Curve of motor output power changing with motor speed different accelerator pedal opening.

**Fig 29:**
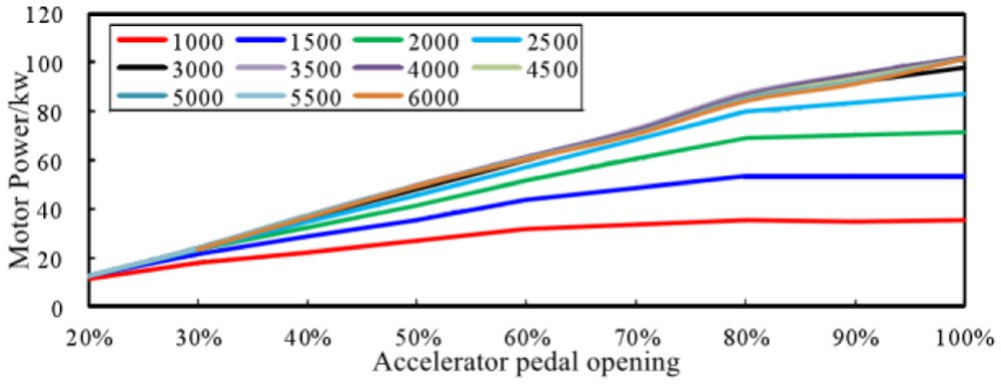
Curve of motor output power changing with accelerator pedal opening under different motor speeds.

According to the test results of motor torque and motor speed, the motor output power under different pedal openings was calculated, as shown in Fig 28. Fig 29 shows the variation curve of motor output power with accelerator pedal opening at different speeds.

**Fig 30:**
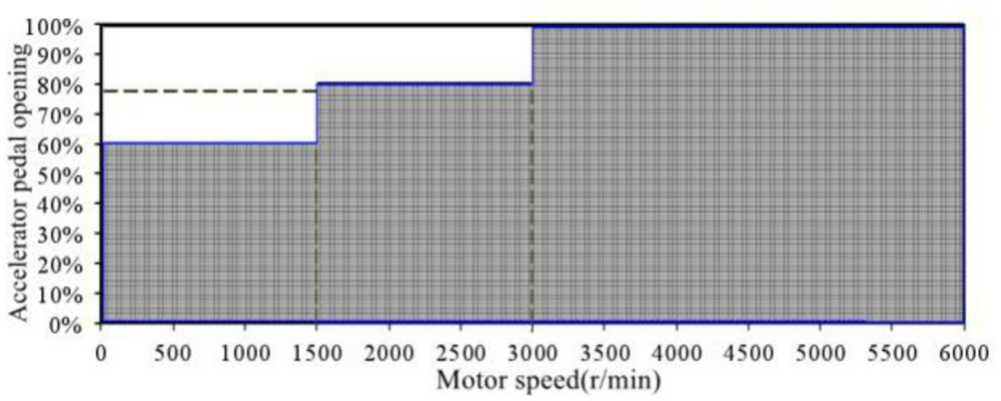
The distribution of the output power of the drive motor linearly varying with the opening of the accelerator pedal.

**Fig 31:**
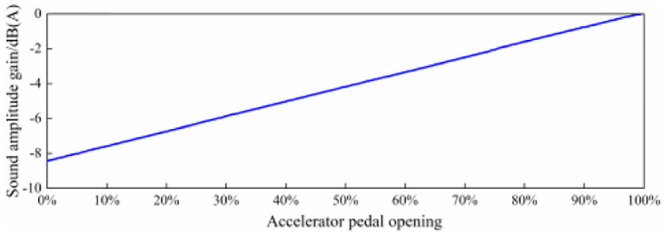
Curve of sound amplitude gain with accelerator pedal opening.

Based on the analysis results of the motor output power, the linear variation distribution of the output power of the drive motor with the openness of the accelerator pedal is summarized. As shown in Fig 30, it can be seen that under most driving conditions of the EV, the output power of the drive motor changes linearly with the openness of the accelerator pedal. When the EV is in the operating condition region as shown in Fig 31, the motor output power changes linearly with the accelerator pedal openness, that is, at the same motor speed, when the accelerator pedal openness increases by Δ*Pedal*, the motor output power increases by Δ*P*_*m*_, and the ratio of the two is constant. Meanwhile, according to the research conclusion in the previous section, there is a linear relationship between the order sound amplitude gain of the engine in conventional vehicles and the engine output power load ratio. Therefore, a sound amplitude gain change curve similar to Fig 31 can be developed to control the sound amplitude of the ASGS in EV.

Since the interior sound positioning of traditional FV used in this article completely conforms to the conceptual design goal of interior sound of the EV, active sound generation control in accordance with this curve can achieve the goal of both comfort and power sound quality characteristics of interior sound of EV. Therefore, according to the actual control situation of the active sound generation control program, the control strategy of sound amplitude gain along with the opening of the accelerator pedal is formulated as shown in Fig 31.

## 5. Development of ASGS for EV

### 5.1. Hardware development of ASGS

**Fig 32:**
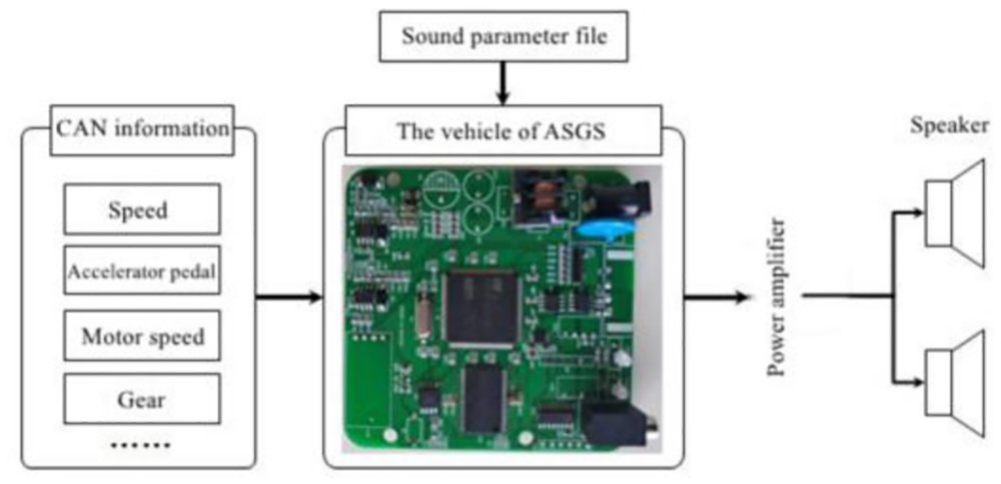
Working principle diagram of ASGS.

Inside the main working principle of ASGS is the real-time control system based on information from vehicles CAN read from the speed, the engine speed (or motor speed), the accelerator pedal opening, gear and other information, and connecting with the voice of design parameters of the documents in advance, the real-time computing synthesis current conditions corresponding voice signal, then through the power amplifier output to the vehicle sound system. In Fig 32, the working principle of the system is shown.

**Fig 33:**
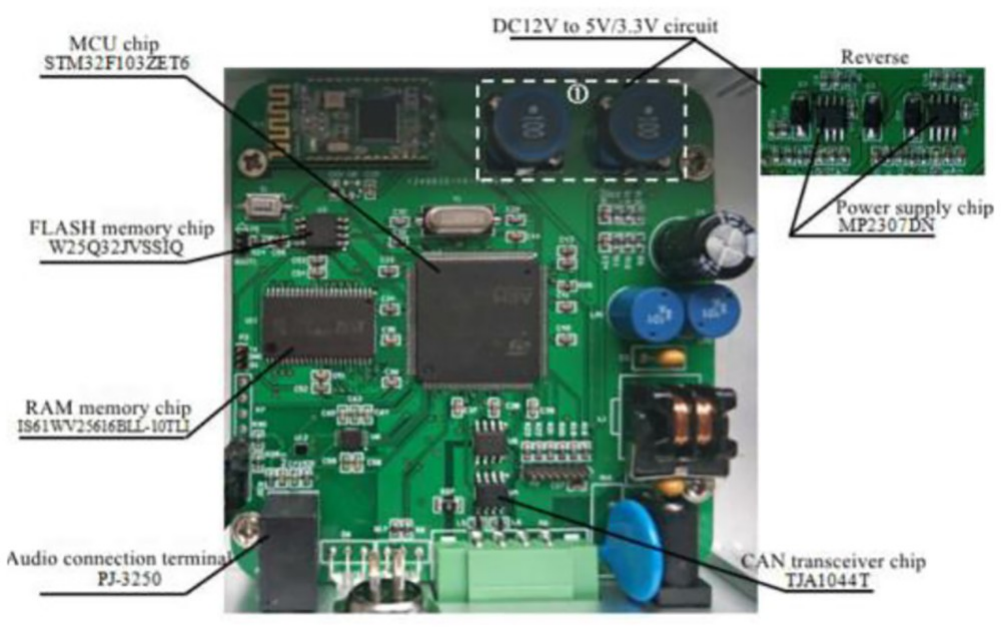
The actual circuit board of ASGS.

**Fig 34:**
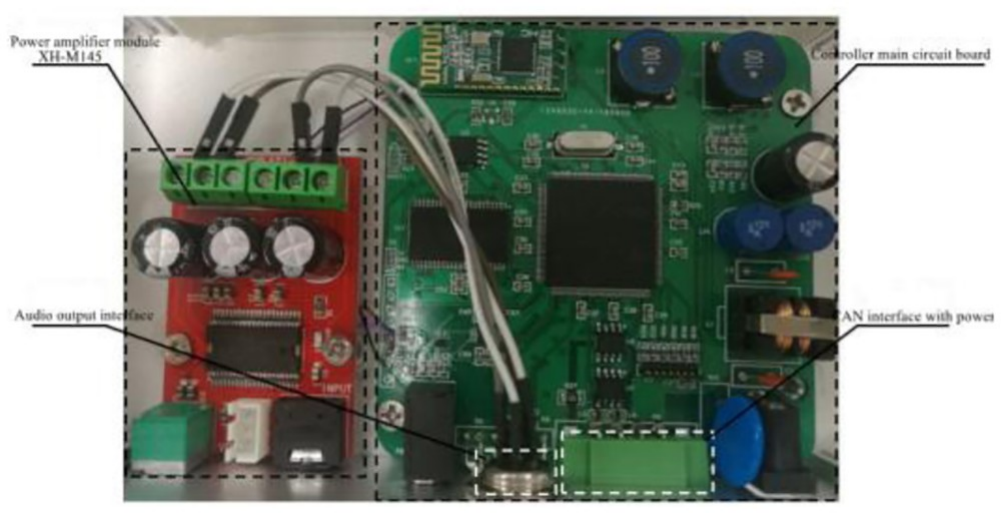
The complete controller of ASGS.

The actual circuit board is shown in Fig 33, and the complete controller is shown in Fig 34.

### 5.2. Software development of ASGS

On the basis of completing the hardware design and development of the control system, the C language is used to write the software of the ASGS in the EV. In order to ensure a clear structure and operability of debugging, the software program is modularized and divided into the following modules: Chip initialization module, CAN signal data acquisition and processing module, FLASH sound data reading and processing module, sound real-time calculation and output module, etc.

**Fig 35:**
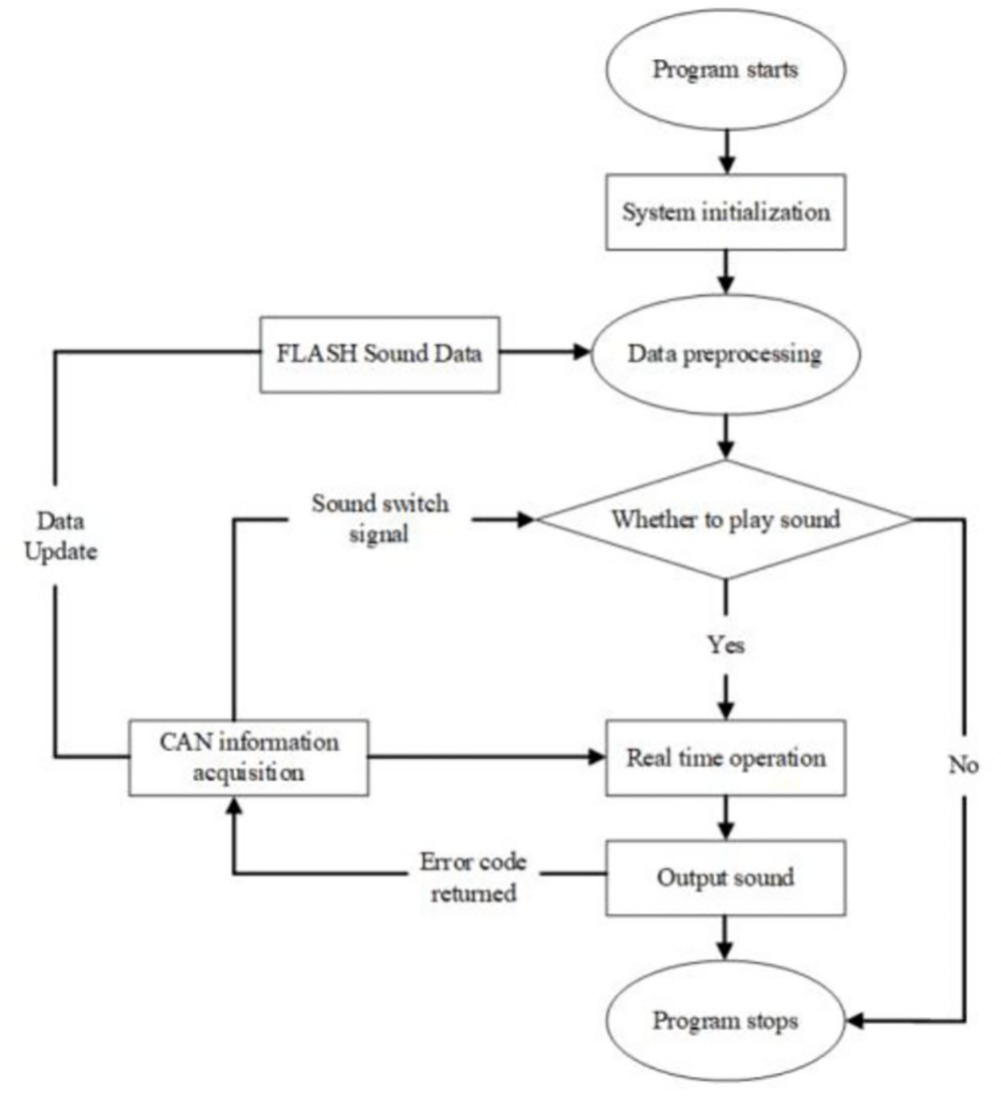
The flow chart of the control software of ASGS.

The flow chart of the control software of ASGS is shown in Fig 35. The specific process is as follows: after the program is started, the system is initialized. On the one hand, the variable in the active voice control program is defined; on the other hand, the constant quantity and constant quantity matrix in the control program are as-signed according to the preset main parameters. Among them, the variation mainly includes the speed V (since the ratio of motor speed *RPM*_*Motor*_ to V is constant, the control program only needs to choose one of the two for calculation), acceleration pedal opening, gear, etc. Constant quantity mainly includes initial virtual engine speed *RPM*_*VEO*_, ratio coefficient *k* between virtual engine speed *RPM*_*VEO*_ and V, number of engine order components *K*, etc.

The sound data reading and pretreatment module calculates the virtual engine speed *RPM*_*VE*1_ according to the vehicle speed *V*_1_ under the current working condition obtained from CAN information, reads the sound amplitude and phase of each order frequency of the engine sound corresponding to *RPM*_*VE*1_, and pre-synthesizes the corresponding engine sound under the virtual engine speed. Then judge whether the current control program is allowed to play the sound. If not, stop the program directly and do not play the sound. If playing is allowed, the real-time sound calculation and output module will read the engine sound corresponding to *RPM*_*VE*1_, accelerator pedal opening *Pedal* and *V*_1_ and other parameters according to the sound data reading and the pre-processing module output. Thus, the sound amplitude correction gain *SPL*_*Gain_p*1_ corresponding to *Pedal*_1_ and *SPL*_*Gain_V*1_ corresponding to *V*_1_ are calculated, and the sound data are converted into analog sound signals through D/A, and finally transmitted to the vehicle sound system speakers through the audio output interface. ASGS play the engine sound corresponding to *V*_1_ and *Pedal*_1_ of the current vehicle driving condition.

### 5.3. Construction of ASGS in EV

#### 5.3.1. CAN communication of the vehicle

The vehicle CAN OBD interface of the EV selected in this article is defined as shown in Table 6, in which pin 1 and pin 9 CAN provide the required information of vehicle speed, motor speed and accelerator pedal position for the ASGS in the EV. By importing the vehicle CAN BUS DBC file to analyze the CAN information, vehicle speed, motor speed and accelerator pedal position signal information is shown in Table 7, so as to ensure that the control system can normally read the required information in the process of working.

**Table 6:** Definition of a certain pure electric SUV OBD interface.

Pin	1	2	3	4	5	6	7	8
Interface definition	Power CAN_H	EV CAN_H	Expand CAN_H	Chassis	Signal	Diagnosis	Reserved	Signal CAN_H
Pin	9	10	11	12	13	14	15	16
Interface definition	Power CAN_L	EV CAN_L	Expand CAN_L	Chassis CAN_H	Chassis CAN_L	Diagnosis CAN_L	Signal CAN_L	Power

**Table 7:** ID information of main parameters in electric vehicle CAN bus.

Signal Name	BUS Name	Mseeage Name	Mseeage ID	Signal Name
Accelerator pedal position	CAN 1	HCU_4	0×122	HCU_ Acceleration pedal position
Vehicle speed	CAN 1	ABS_1	0×B4	ABS_ Vehicle speed
Engine speed	CAN 1	HCU_18	0×149	MCU1_TM speed

#### 5.3.2. Construction of equipment

**Fig 36:**
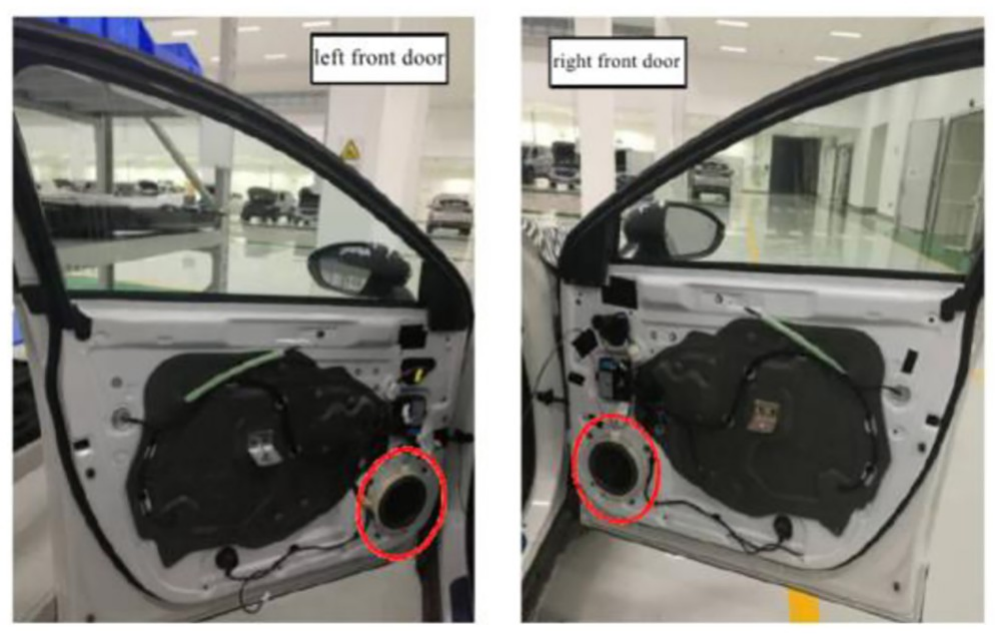
The placement of the mid-bass speakers.

**Fig 37:**
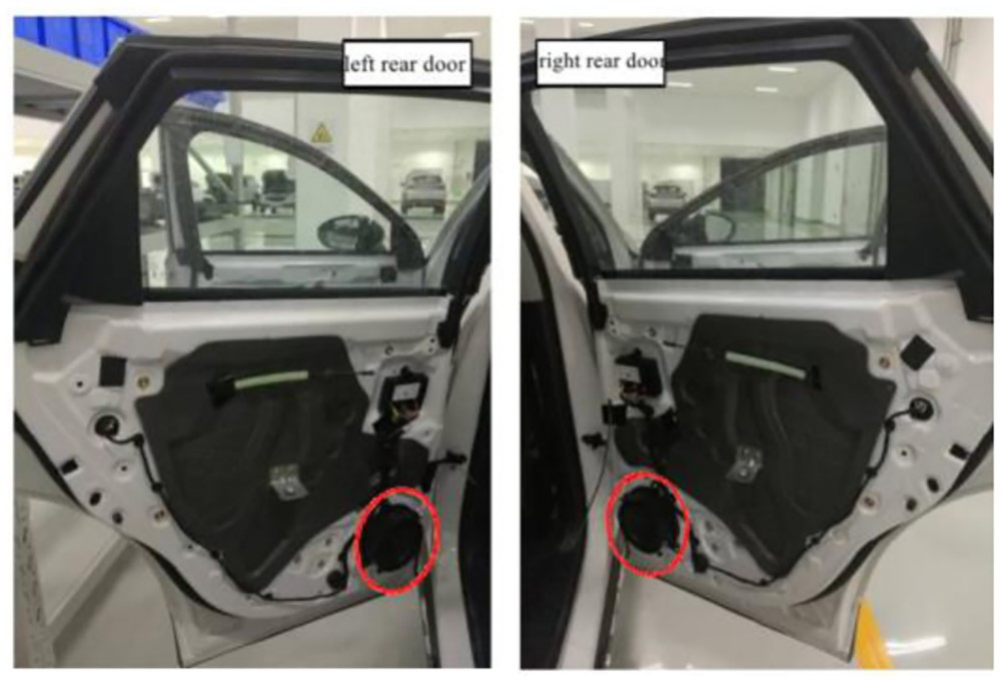
The placement of the full-range speaker.

**Fig 38:**
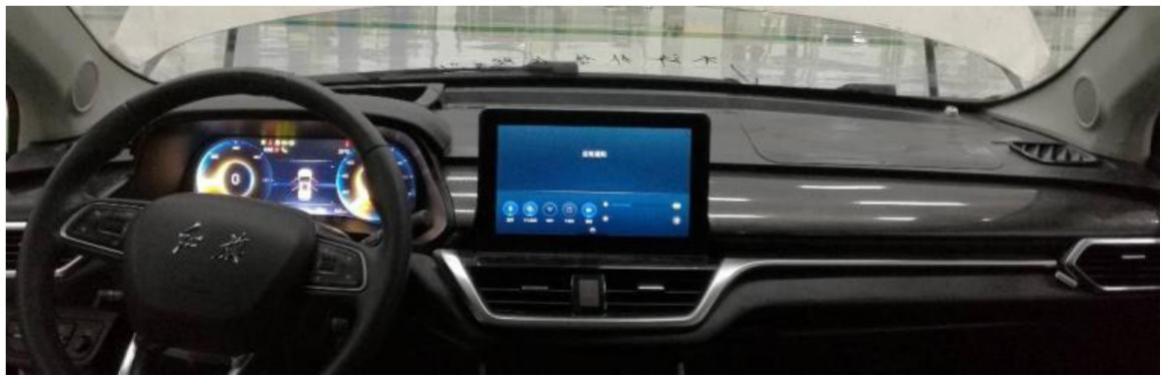
The placement of the tweeter.

The composition and layout of the Hi-Fi sound system of the EV are as follows: (1) Power amplifier: The power amplifier of the sound system is arranged in the trunk, and is connected to six speakers through the wire harness. (2) Loudspeaker: two low-frequency loudspeakers are respectively arranged in the lower right corner of the left front door and the lower left corner of the right front door, as shown in Fig 36, two full-frequency loudspeakers are respectively arranged in the lower right corner of the left rear door and the lower left corner of the right rear door, as shown in Fig 37, Two high-frequency loudspeakers are respectively arranged at the junction between left and right Pillar A and instrument panel, as shown in Fig 38.

## 6. Test and analysis

### 6.1. Verification of accuracy of ASGS

In order to verify the sound synthesis accuracy of the ASGS, the final sound scheme of the accelerated vehicle in section 3.2 was discretized, and the frequency, amplitude and phase of each engine order component were obtained through STFT and imported into the controller. When the controller received the engine speed signal, The program of ASGS receives the corresponding sound parameter information to synthesize the engine order sound, and then determines the overall sound amplitude according to the current virtual accelerator pedal opening information and vehicle speed information, and finally realizes sound playback through the Hi-Fi speaker.

**Fig 39:**
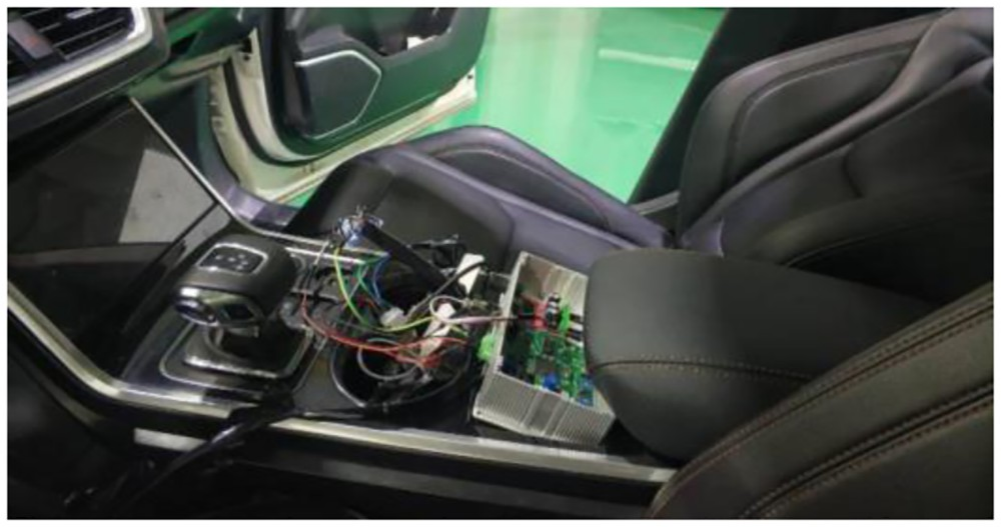
The layout of frequency response test controller of vehicle audio speaker.

The EV studied in this article is parked in the semi-anechoic room of the vehicle, and the ASGS is built to ensure that the controller can transmit sound signals to the on-board audio speakers through its audio output interface, as shown in Fig 39. The signal acquisition method is the same as that in section 2.1.

Set the input signal of virtual engine speed to the same working condition as scheme 3 in section 3.2, that is, when the virtual accelerator pedal opening is 100%, the engine speed accelerates from 1000 r/min to 6000 r/min. Adjust the volume control knob of the Hi-Fi speaker to a position corresponding to the sound amplitude of the design state after adjustment in scheme 3 in Fig 21. Set the virtual engine speed input signal to the same working condition as scheme 3 in Section 3.2, that is, the engine speed accelerates from 1000 r/min to 6000 r/min, and the virtual accelerator pedal opening is 100%. Adjust the volume control knob of the Hi-Fi speaker to a position corresponding to the sound amplitude of the design state after adjustment in scheme 3 in Fig 22.

**Fig 40:**
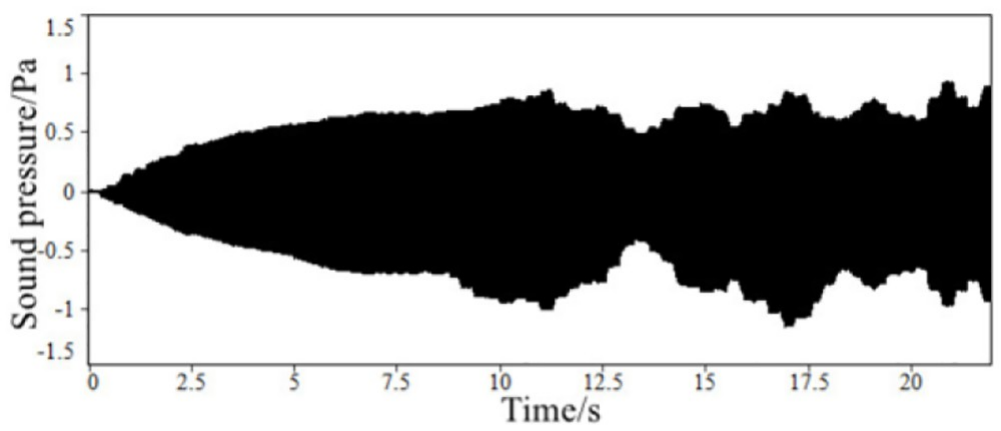
The test time-domain signal of the simulated acceleration driving sound.

**Fig 41:**
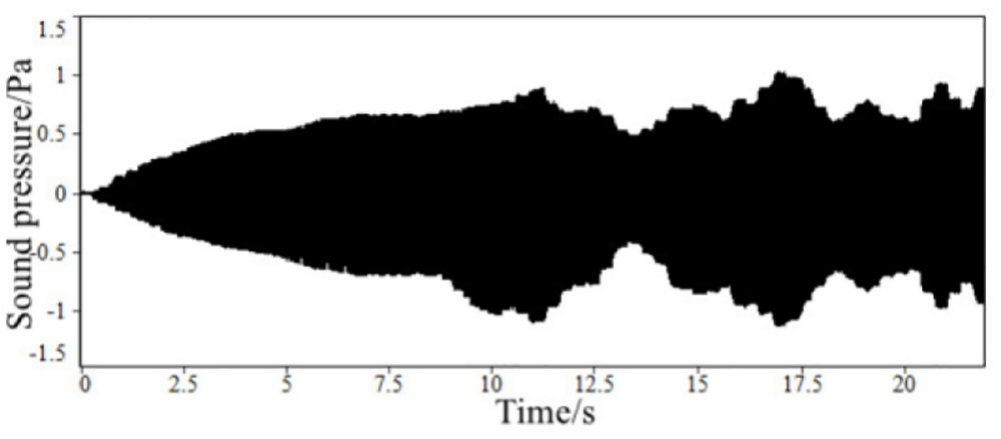
The time-domain signal of scheme three.

**Fig 42:**
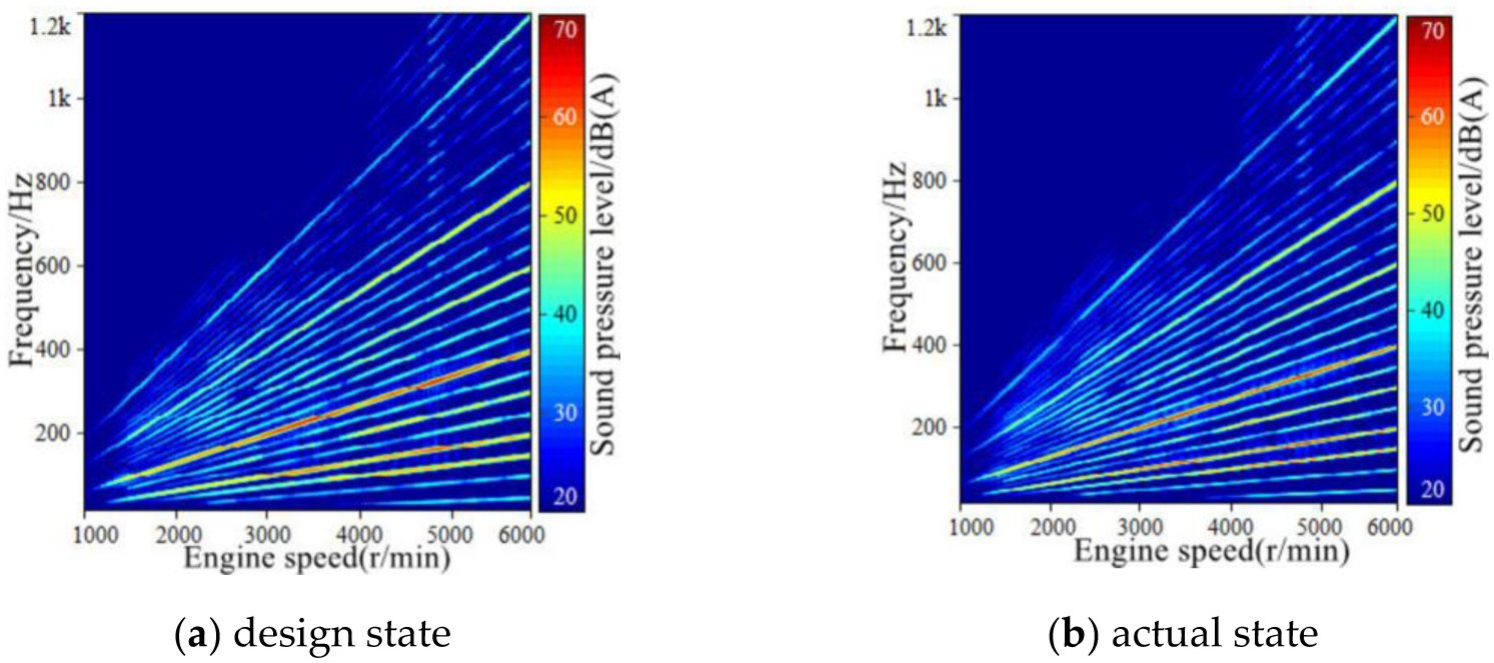
FFT spectrum comparison of two states of sound with the change of virtual engine speed.

In order to facilitate the comparison of engine sound fitting precision, test the sound signal generated by the speaker in the process of virtual engine speed change of 1000–6000 r/min. The time domain signal of the sound in the simulated accelerated driving vehicle obtained through the high-fidelity speaker playback test is shown in Fig 40. The original time domain signal of the sound design in the accelerated driving vehicle in scheme 3 is shown in Fig 41, and the spectrum comparison is shown in Fig 42.

The results show that the time-domain signals of the two states are basically consistent, but are slightly different in the range of 15–18 s. The subjective evaluation of the two states of engine sound has a good consistency. From the comparison of the spectrum diagram, the sound synthesized by the ASGS can truly restore the sound spectrum characteristics of the design state from three dimensions: engine order composition, energy distribution in the frequency domain and sound amplitude enhancement in the typical speed range. Therefore, the engine sound simulation precision of the ASGS is high, that is, the simulation precision of the engine order sound can meet the requirements of the system design.

### 6.2. Calibration of ASGS sound amplitude and verification of target achievement

On the basis of the above section, the calibration of simulated sound amplitude is carried out, and the main parameters are as follows:

①The virtual engine speed simulates the acceleration condition, accelerating from 1000 r/min to 6000 r/min.

②The virtual accelerator pedal opening is set to: 20%, 30%, 40%, 50%, 60%, 70%, 80%, 90%, 100%;

③The accelerator pedal opening gain is set to the parameter state in Fig 32, that is, the corresponding sound amplitude gain at 20% opening is a-6.8dB(A), and the sound amplitude gain at 100% opening is 0 dB(A).

④Adjust the power amplifier adjustment knob of the Hi-Fi speaker so that the sound amplitude at 100% opening is consistent with the total sound value of the adjusted state in scheme 3 in Fig 22.

**Fig 43:**
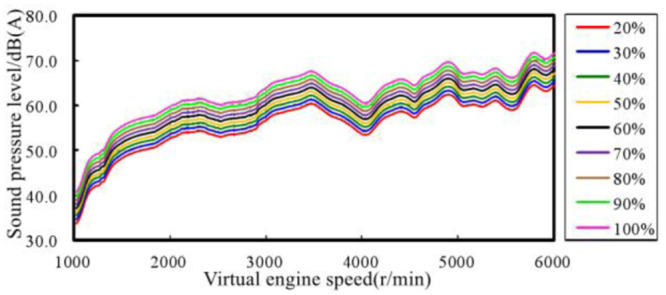
Variation curve of sound amplitude of active sound system with virtual engine speed.

As the setting of sound amplitude gain along with the speed control curve needs to be combined with the original background sound inside the EV under real vehicle driving conditions, this parameter is not set here. Therefore, according to the above parameter settings, the sound signal of the Hi-Fi speaker measurement point was tested under the conditions of 20%, 30%, 40%, 50%, 60%, 70%, 80%, 90% and 100% of the virtual accelerator pedal opening, and then the change of the total sound value with the virtual engine speed under each condition was calculated, as shown in Fig 43.

**Fig 44:**
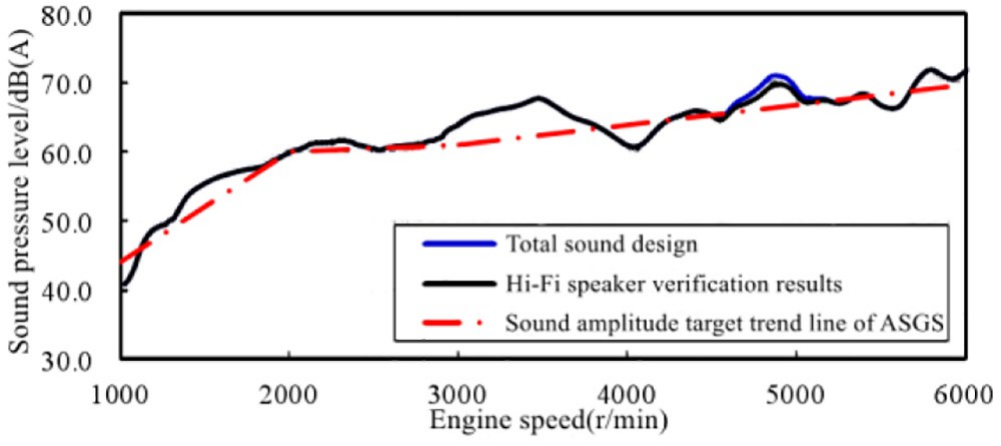
The verification of the virtual calibration target of the acceleration driving sound amplitude under 100% accelerator pedal opening.

Under the condition of Hi-Fi speaker, the acceleration driving condition of 100% accelerator pedal opening is simulated. The achievement of the sound amplitude target of the system is shown in Fig 44. It can be seen that the total sound test results under this condition are in good agreement with the total sound curve of the design scheme. The variation trend of sound amplitude produced by the system through the high-fidelity loudspeaker is consistent with the target trend line, and there is a slight difference in the speed range of 4500–5300 r/min. There is an error of 1.4 dB(A) between the sound amplitude of the system and the design state near 4900 r/min.

It can be seen from the above that under the simulated acceleration driving condition with 100% accelerator pedal opening, the sound amplitude variation produced by the ASGS through the Hi-Fi loudspeaker is in good agreement with the sound amplitude variation of the design state, with high sound amplitude control accuracy and sound amplitude error controlled within 1.4 dB(A). And the overall trend change of sound is in line with the trend line of sound amplitude target.

### 6.3. Test of in-vehicle sound of EV’s ASGS

**Fig 45:**
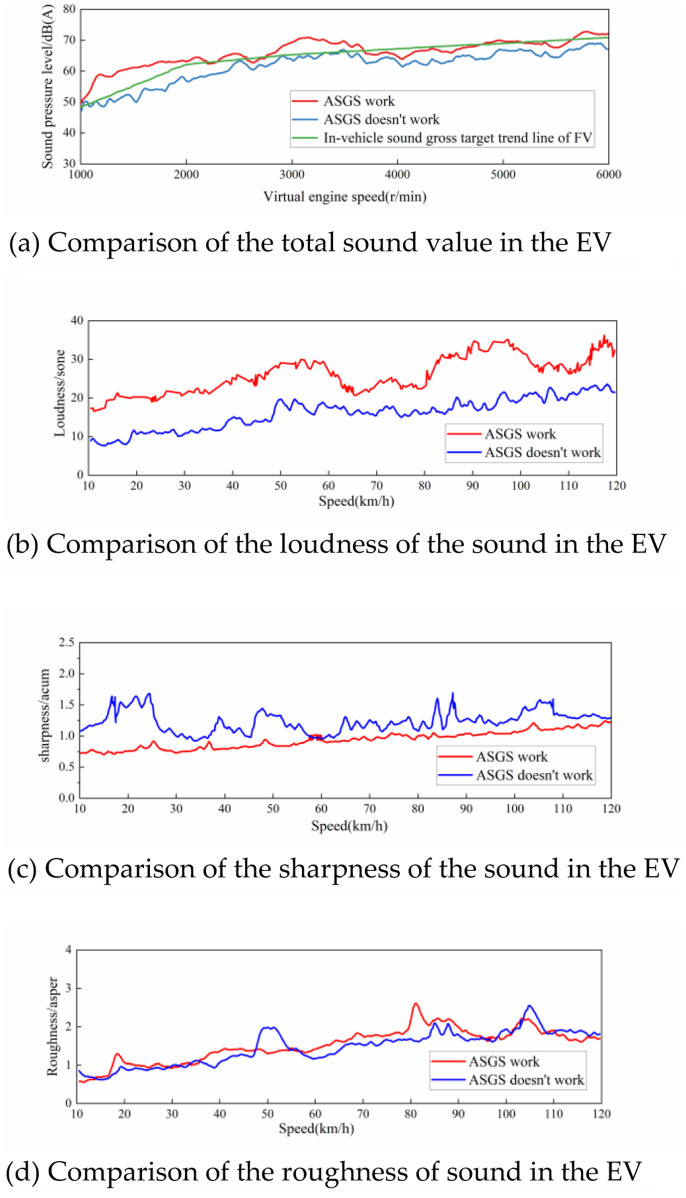
Comparison of interior sound in two states.

Under both working and non-working states of the ASGS, the sound signal of the driver’s right ear position in the vehicle during acceleration under 100% accelerator pedal openness was tested, and the sound situation inside the vehicle under working and non-working states of the ASGS was calculated respectively, as shown in Fig 45.

## 7. Conclusions

This article analyzes the interior sound spectrum characteristics of traditional FVs and EVs during acceleration, and verifies the accuracy of the short-time Fourier trans-form and synthesis technology based on Kaiser window function for the extraction and synthesis of engine order sounds. And verifies the correctness of engine sound simulation of ASGS in EV. Based on objective and subjective evaluations, a plan to improve the interior sound quality of EVs during acceleration is proposed, an ASGS is developed, and comparative analysis and verification are carried out based on simulation analysis and real vehicle testing.

This paper develops an active sounding system for EV. The proposed control system and method are capable of active control based on real-time conditions and calibrated to correct the input and output errors of the control system. The final test application shows that the method proposed in this paper is simple, feasible and effective compared with the current technical level.

1. The sound orders in the FV and EV were compared and analyzed, and the sound signal of the engine order in the FV was tested, extracted, synthesized and verified. The idea of simulating the engine order sound in an electric vehicle is established to improve the sound quality.
2. The influence of the engine order composition and the energy distribution in the frequency domain on the interior sound quality is analyzed, and the local enhancement area of the sound amplitude in the vehicle speed domain is determined. Combined with the sound quality characteristics of a coupe-type FV, the specific acceleration sound design requirements of the EV, the total interior sound target and the simulated engine sound target are determined.
3. Completed the software and hardware development of the active control sound system, built the active sound system in the EV based on the original hi-fi system, and carried out the sound synthesis accuracy test. The results show that the designed active sound system has good sound synthesis accuracy and high reliability
4. The real vehicle test was carried out to verify the achievement of the sound target when the active sound system works normally. Among them, the achievement of the acceleration driving condition target is that the total value of interior sound can meet the requirements of the set target trend line, forming an order architecture spectrum feature dominated by the fourth-order components of the engine and supplemented by other orders, and achieved sound amplitude enhancement in the range of 40–75 km/h. The sound pressure level and loudness of interior sound have been in-creased, and the sharpness of the sound inside the car has been improved, with a maximum reduction of 1.0acum.

## Supporting information

S1 File (PDF)
