# Supplementary material for: Research on the characteristics of EV interior sound quality and its dynamic active control system design and development un-der accelerated driving conditions
Source: PLoS One. 2024 Apr 1;19(4):e0290150. doi: 10.1371/journal.pone.0290150 (PMC10984514; doi:10.1371/journal.pone.0290150)
Supplement: S1 File — (PDF) [file pone.0290150.s001.pdf]

This paper develops an active voice control system for electric vehicle. The working principle of the control system is shown in Fig 1.

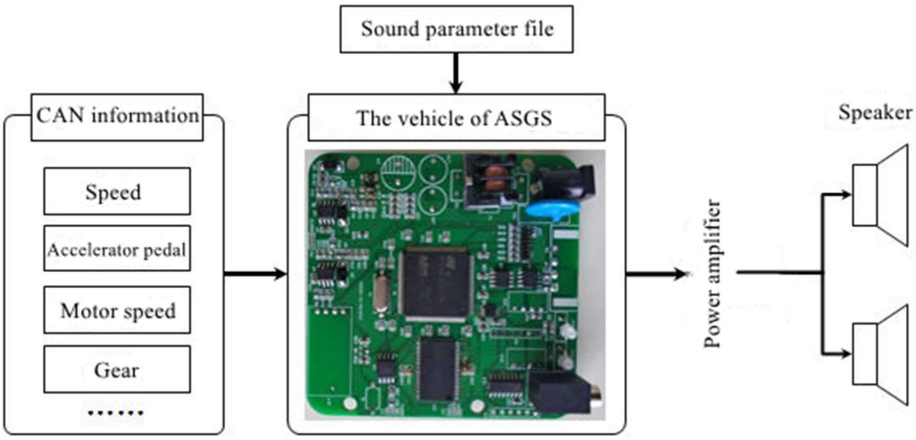

Fig 1. Working principle diagram of ASGS.

The working flow chart of the developed system is shown in Fig 2.

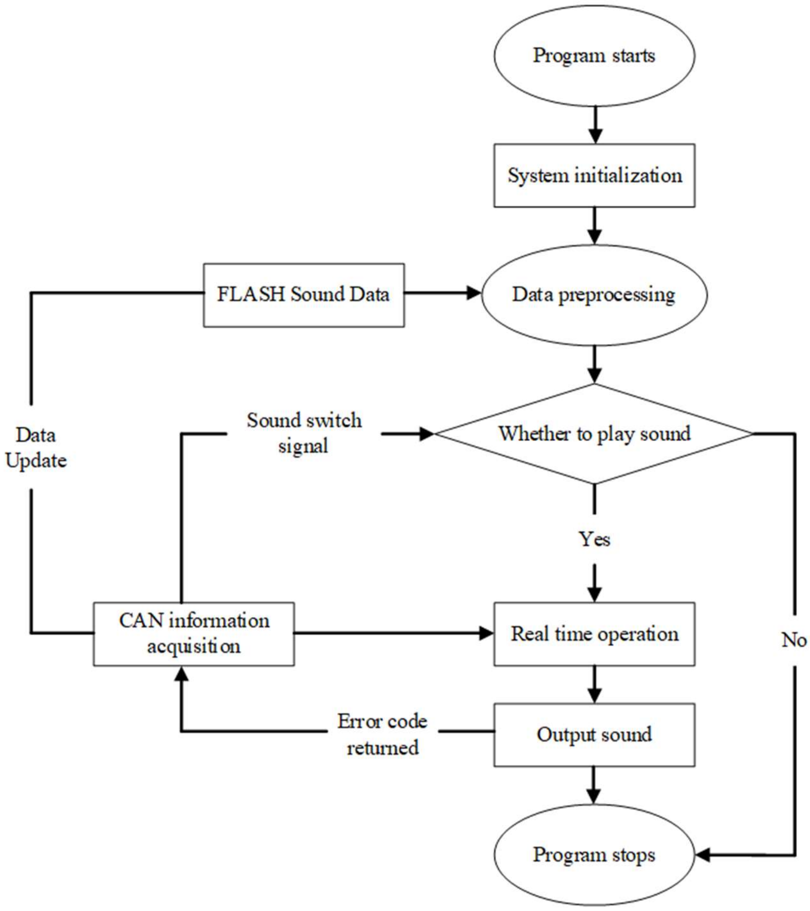

Fig 36. The flow chart of the control software of ASGS.

After the final real vehicle test, the data of the system in two states when it is working and not working are shown in Fig 3.

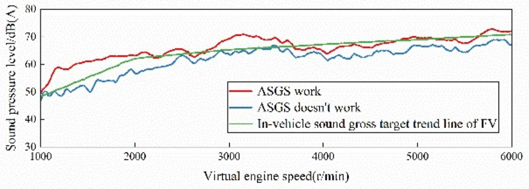

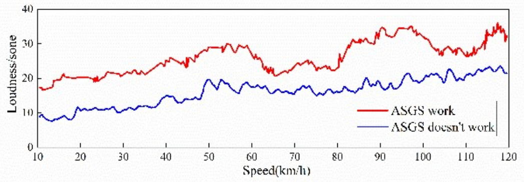

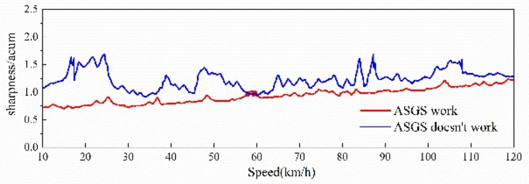

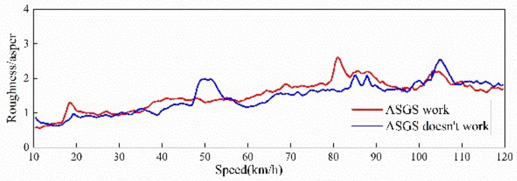

Fig 46. Comparison of interior sound in two states.
